# Numerical modeling of the out-of-plane dynamic response of masonry gable walls via a high-fidelity block-based finite element modeling approach – part II: post-diction and application to other structural configurations

**DOI:** 10.1007/s10518-026-02473-1

**Published:** 2026-04-22

**Authors:** Amirhossein Ghezelbash, Antonio Maria D’Altri, Satyadhrik Sharma, Paulo B. Lourenço, Stefano de Miranda, Francesco Messali

**Affiliations:** 1 Faculty of Civil Engineering and Geosciences, Delft University of Technology, Delft, The Netherlands; 2 Department of Civil, Chemical, Environmental, and Materials Engineering, University of Bologna, Bologna, Italy; 3 Reliable Structures Section, Netherlands Organisation for Applied Scientific Research (TNO), Delft, The Netherlands; 4 ISISE, Department of Civil Engineering, University of Minho, Guimarães, Portugal

**Keywords:** Unreinforced masonry, Numerical modeling, Seismic loading, Out-of-plane, Gable walls, Wall-roof connection

## Abstract

The out-of-plane (OOP) dynamic behavior of unreinforced masonry (URM) gable walls was investigated in this paper using a high-fidelity block-based numerical modeling approach, building on the participation of the authors in the ERIES SUPREME blind prediction competition. In this paper, the numerical models developed for the competition were updated based on the experimental data published after the competition to further improve accuracy. The improvement was obtained by slight recalibration of mortar joint tensile strength and friction between the walls and the loading set-up. The updated models were also adopted to simulate a third wall originally excluded from the competition. The models were then used to complement the experimental campaign with additional configurations in a parametric study. Specifically, the influence of roof-wall connections and pre-existing damage on the performance of the gable walls were examined to address gaps identified in both experimental and numerical studies of the past. Stronger roof-wall connections, while improving global stability and increasing wall OOP strength in the static regime by up to 140%, led to collapse at dynamic loading intensities reduced by an average of 28% and up to a maximum of 57%. This early collapse resulted from the transfer of larger dynamic demands to the gable walls. This higher demand transfer also caused earlier damage initiation and considerable changes in collapse mechanisms, effects not captured by static analysis, highlighting the uncertainties governing dynamic behavior and the need for robust methodologies to address them. Finally, light pre-damage, modelled in this study as a crack at the base of the walls, had only a minor influence on failure mechanisms and OOP resistance.

## Introduction

Unreinforced masonry (URM) gable walls, also referred to as gables or walls in this paper, are triangular walls situated between the topmost floor and the roof in URM buildings (Sorrentino et al. 2016). Failures of roof structures and gable walls have been demonstrated to represent significant vulnerabilities in URM buildings during seismic events, due to their inherent flexibility, minimal overburden, and elevated position within the building where seismic accelerations are typically amplified (Tomassetti et al. 2019; Adhikari and D’Ayala 2020). It has been consistently identified in post-earthquake assessments that these failures are major contributors to life safety risks (Penna et al. 2014; Dizhur et al. 2016). Moreover, the out-of-plane (OOP) behavior of URM gable walls has remained a critical challenge in earthquake engineering, particularly due to their sensitivity to boundary conditions, construction details, and initial imperfections (Sorrentino et al. 2016; D’Ayala and Paganoni 2011). Despite this inherent vulnerability of URM gables (Penna et al. 2014; D’Ayala and Paganoni 2011; Moon et al. 2014; Page 1991; Oyarzo-Vera and Griffith 2009) and the lack of knowledge on their seismic behavior, little attention has been given to the study of these walls in laboratory settings due to financial and time constraints, as well as limitations of the test set-ups (Tondelli et al. 2016; Penner and Elwood 2016; Sharma et al. 2021; Tomassetti et al. 2019). Moreover, URM gable walls have been somewhat overlooked for individual study in numerical investigations, due to the lack of benchmark experiments to validate modeling approaches. URM gable walls have been simulated individually only in a few contributions (Canditone et al. 2025; Malomo and DeJong 2022), while they are considered as part of a larger building system in others (Tomić et al. 2021; Davis et al. 2024; Guerrini et al. 2023). Globally, various numerical approaches, from simplified macro-element (Malomo and DeJong 2022; Vanin et al. 2020; Kesavan and Menon 2023) and continuum-based (Lourenço 2000; Noor-E-Khuda 2021; Noor-E-Khuda et al. 2016; Selby and Vecchio 1993; Rots et al. 2016), to multi-scale (Sharma et al. 2021) and detailed block-based (Mohyeddin et al. 2013; Kesavan and Menon 2022; D’Altri et al. 2018; Xie et al. 2021; Oktiovan et al. 2024; Macorini and Izzuddin 2011; Nie et al. 2023; Malomo et al. 2020; Oktiovan et al. 2024; Furiosi et al. 2025) ones, have been developed for modeling the dynamics of URM.

In response to the critical need for a deeper understanding of the seismic OOP behavior of URM gable walls, the Seismic oUt-of-Plane Response of Masonry Gables (SUPREME) project was initiated under the EU-funded Engineering Research Infrastructure for European Synergies (ERIES) transnational access program (2022). This comprehensive research campaign, known as ERIES SUPREME, was conducted at the EUCENTRE Foundation in Pavia, Italy. The goal was to evaluate the seismic performance of full-scale URM gable walls via multi-step incremental dynamic shake table tests. Different boundary motions were used to simulate wall interactions with diaphragms of differing stiffness (Sharma et al. 2025). As part of the ERIES SUPREME project, a blind prediction competition was organized. The participants were offered a unique opportunity to benchmark numerical modeling strategies against these full-scale dynamic tests.

In the previous contribution of the authors to the blind prediction competition (Ghezelbash et al. 2026), a high-fidelity implicit 3D block-based Finite Element numerical modeling approach (Ghezelbash et al. 2025; D’Altri et al. 2019) was used to replicate the behavior of two of the walls tested within the ERIES SUPREME experimental campaign (Sharma et al. 2025). Despite limited access to experimental data during the competition, the authors predicted with good accuracy key aspects of the experimentally recorded behavior, including dynamic modal response, failure mechanisms, loading intensities corresponding to collapse, displacement demands, and hysteretic energy dissipation. Central to the blind prediction study was the recognition that dynamic structural response, especially in systems with pronounced material and geometric nonlinearity, is inherently sensitive and dependent on many known and unknown variables. With minor variations in mechanical properties, loading conditions, or connection details, considerably different qualitative and quantitative outcomes, particularly in the near-collapse phase of the response, were observed.

In the present study, the authors build on their blind prediction efforts (Ghezelbash et al. 2026) by entering a post-diction phase. The complete experimental results, made available after the competition, were used to update the high-fidelity numerical models developed during the blind-prediction phase and conduct a deeper investigation on the OOP response of URM gable walls. With the focus shifted from replicating to understanding the OOP dynamic response, the updated models were used specifically to study other configurations, such as strong wall-to-roof connections and pre-existing damage, on the behavior of URM gables. In several studies (Chen et al. 2021; Landi et al. 2015), it has been shown that roof-wall connections, or purlin-wall connections in this case, significantly influence the OOP dynamic behavior of URM structures. The use of strong connections, such as in retrofitted systems where the purlins are bolted or anchored to the walls, enhances wall stability by facilitating effective load transfer and, expectedly, reducing displacement demands (Tomassetti et al. 2019; Penner and Elwood 2016). On the other hand, the use of inadequate or flexible connections can lead to large OOP displacements and wall instability (Tomassetti et al. 2019; Penner and Elwood 2016). That said, the influence of different connection configurations on the response of URM gables has not been consistently investigated in any numerical or experimental campaign. It is also noted in the literature (Dolatshahi and Aref 2015) that the presence of pre-damage caused by prior loading history or structural defects can significantly reduce the load-bearing capacity and alter the failure mechanisms of URM constructions. Nevertheless, engineers often operate under the assumption that structures are in pristine condition, neglecting the potential impact of existing damage (Grillanda et al. 2021). This oversight can result in less accurate assessments of structural capacity and safety (Bureau of Indian Standards 1987; Standards Australia Committee BD-004 2011; European Committee for Standardization 2005; Netherlands Standardization Institute 2020; Masonry Standards Joint Committee (MSJC)).

The paper has been structured as follows. A summary of ERIES SUPREME experiments (Sharma et al. 2025) has been provided in Sect.  2, with a description of how the blind prediction numerical models were updated to reproduce the experimentally observed behaviors with greater accuracy. The results of post-diction numerical simulations have been presented in Sect.  3 and compared against experimental outcomes. Here, the modelling efforts were also extended to the third wall tested in the ERIES SUPREME campaign (Sharma et al. 2025). This wall had not been originally included in the competition and loading conditions in-between the two walls simulated during blind-prediction phase. In Sect.  4, the parametric study conducted via the updated numerical models for exploring alternative purlin-wall connection configurations and pre-damage scenarios has been described, along with the interpretation of its outcomes. Finally, the concluding remarks and the findings of the paper have been presented in Sect.  5, with recommendations for future research. By this research, the authors provide broader insights into seismic vulnerability and mitigation strategies for URM gables with complex geometries and boundary conditions.

## Updated information regarding the experimental campaign and the numerical models

An overview of the specimens tested in the ERIES SUPREME experimental campaign (Sharma et al. 2025) has been presented in this section, along with the past numerical models developed by the authors. To maintain brevity, detailed descriptions have been omitted, focusing instead on a concise summary of the tests, the description of the new experimental data made available after the competition, and the changes made to the numerical models in the post-diction phase. Readers may refer to (Sharma et al. 2025; Ghezelbash et al. 2026) for a more comprehensive description of the tests and the numerical modeling approach adopted in this study.

### Summary of the experimental set-up and tested wall specimens

**Fig. 1:**
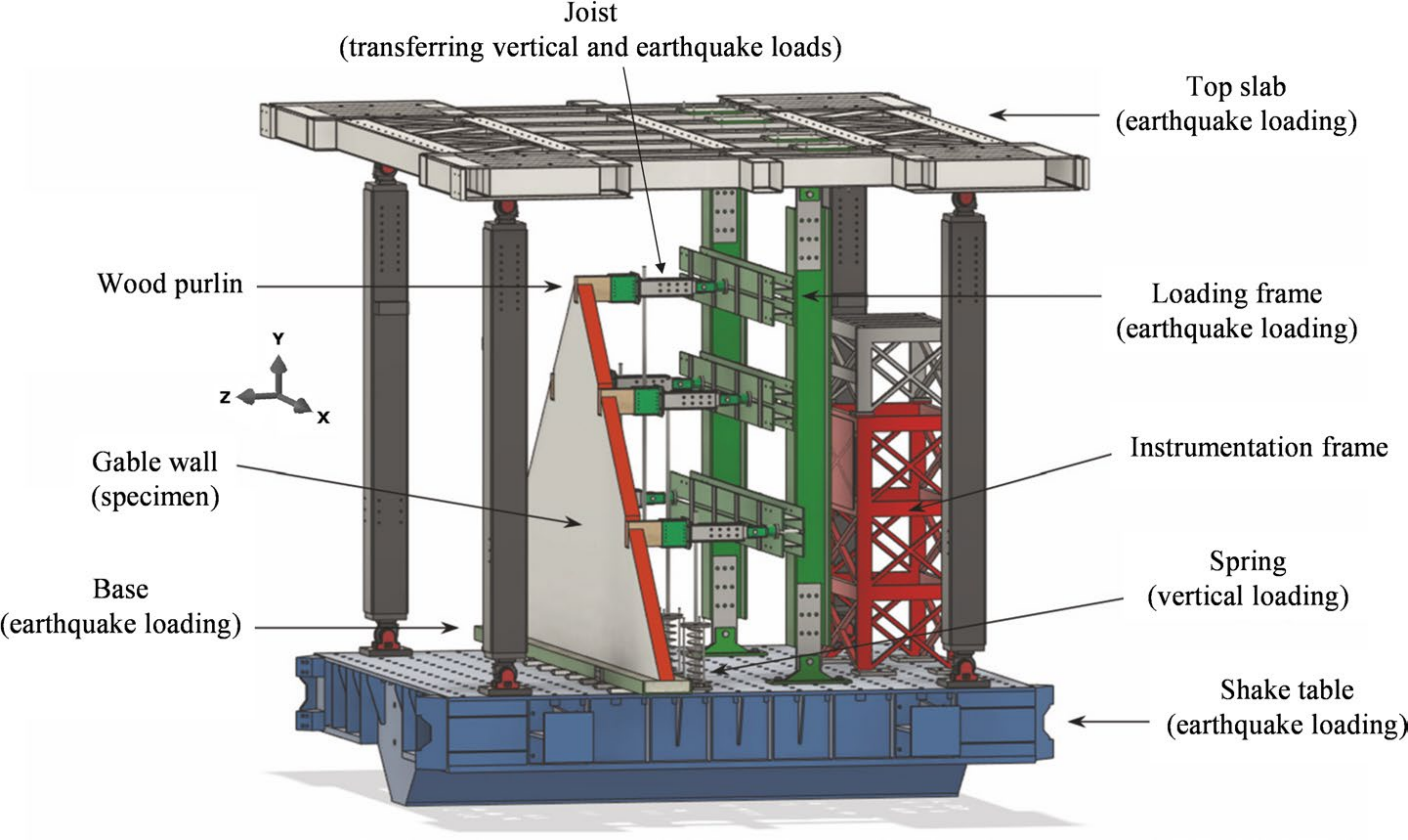
Overview of the experimental set-up used to test the gable walls, adapted from (Sharma et al. 2025)

Three single-leaf unreinforced masonry gable walls had been constructed using clay bricks and lime-based mortars in a stretcher bond pattern, and had been tested in (Sharma et al. 2025) using the setup shown in Fig. 1. Each wall was an isosceles triangle, built on a regular mortar bed joint placed over a pre-stressed reinforced concrete foundation. The foundation had been bolted to a shake table. The top motion had been applied to a steel slab supported by four vertical steel columns. The motion had been transferred to the wall via a steel frame and joist system. The frame had been constructed via two steel columns connected by three rows of horizontal beams, with steel joists linking the beams to timber purlins. The purlins, located at three different elevations, had been housed in dry-friction joist pockets (referred to as wedges here) cut into the walls. Each wedge had accommodated one purlin via a dry mortar layer beneath the purlin while leaving gaps along the vertical edges. Vertical loads had been introduced through rod and spring assemblies connected to the joists at an offset from the walls. Cylindrical hinges had been included in the setup at critical connection points (between the top slab and columns, the shake table and columns, and the beams and joists), allowing rotation around the in-plane axis as necessary. The connections between the steel profiles of the loading frame, the joists, and between the steel joists and timber purlins had been designed to behave rigidly, minimizing relative movement of these components and ensuring consistent transmission of dynamic loads from the shake table to all elevations of the walls.

### Final experimental seismic input motions and loading sequence

**Fig. 2:**
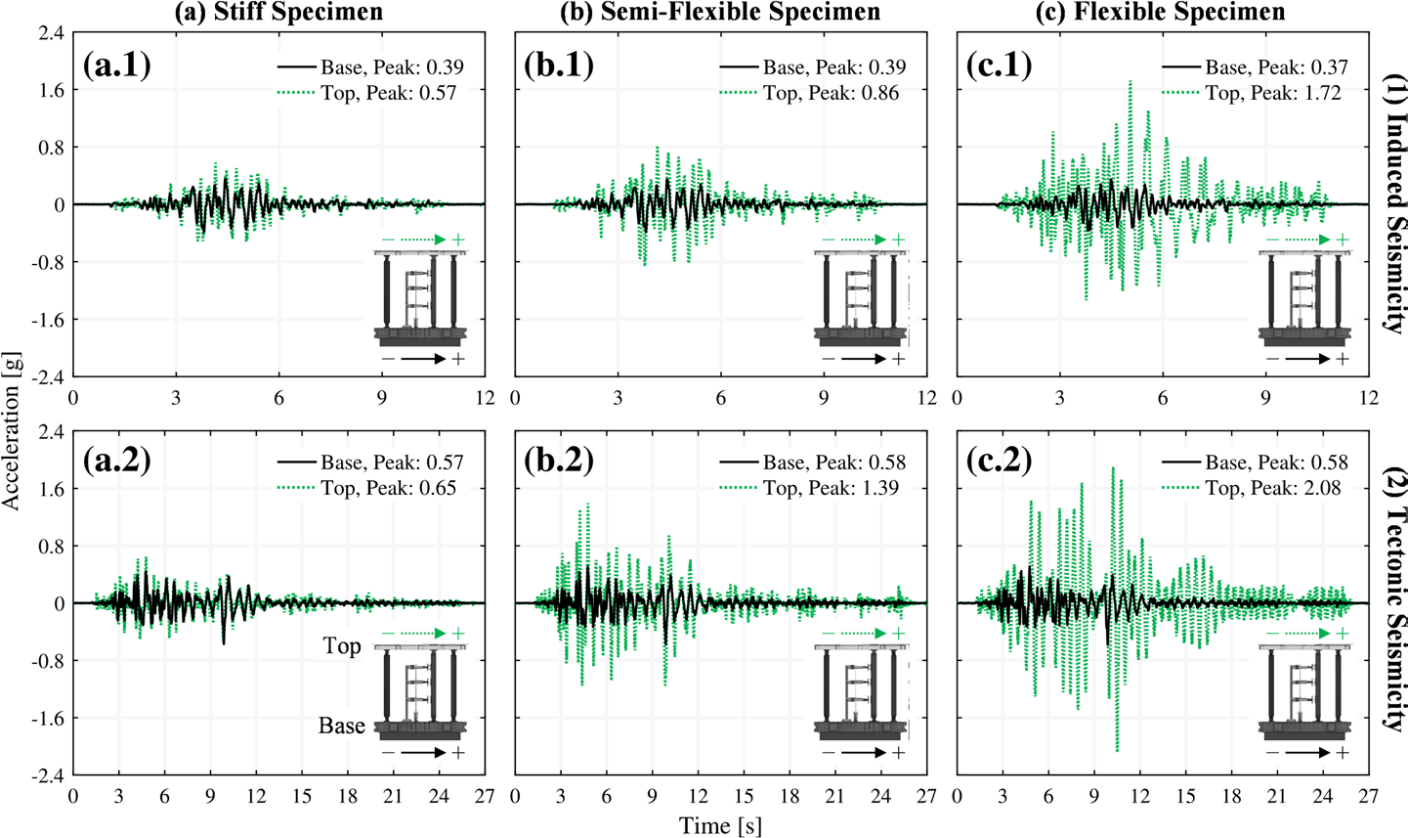
Dynamic motions recorded during the shake table tests

According to the experimental data published after the competition, each specimen had been subjected to a 4.5 kN (an update from the 4 kN initially reported in the prediction phase) vertical load at each purlin-wall connection, and then subjected to multi-step dynamic loading sequences via the input signals shown in Fig. 2. Each wall test had been conducted using two sets of acceleration time histories: one representing induced seismicity and the other tectonic seismicity. The induced seismicity signals, shown in Fig. 2.x.1, had been derived from a numerical model of a typical Groningen terraced house in the Netherlands (Mirra et al. 2020), subjected to earthquakes associated with gas extraction activities in that region (Bommer et al. 2015). The tectonic signals, shown in Fig. 2.x.2, had been obtained from a single-degree-of-freedom model of the same building, using accelerograms recorded during a central Italy earthquake (D’Altri et al. 2018). More information regarding the loading signals can be found in (Mirra et al. 2025).

Each acceleration time history pair consisted of one signal applied to the shake table and one to the top slab. One wall (referred to as the “Stiff” specimen or the case under “Equal” boundary motions) had been subjected to theoretically identical motions at its top and bottom, as shown in Fig. 2.a, representing stiff roof diaphragms. Although, minor discrepancies had been observed due to challenges in maintaining identical top and bottom motions in experimental settings. To simulate the effects of flexible roof diaphragms, the second wall (referred to as the “Flexible” specimen or the case under “Differential” boundary motions) had been tested with two considerably different signals in each pair, as depicted in Fig. 2.c. In this second test, the top signal had been out-of-phase and magnified in amplitude with respect to the base signal. The third wall, representative of semi-flexible retrofitted diaphragms (referred to as the “Semi-Flexible” specimen or the case under “Semi-Differential” boundary motions), had been subjected to signals with intermediate differences in each pair, as shown in Fig. 2.b. In this third test, the top signal had only been amplified in magnitude while remaining in phase with respect to the base signal. It should be noted that during the blind prediction, only sample signal pairs had been published to be scaled up and down for the induced and tectonic seismicity loading of each wall. The acceleration time histories recorded from the shake table and top slab during all loading runs of the three experiments were made available after the competition.

The loading signals presented in Fig. 2 had been applied as acceleration time histories to the specimen boundaries according to the sequences outlined in Table 1. Six loading runs using induced-seismicity signals, followed by additional runs with tectonic-seismicity signals, had been included in each test. The loading intensity had been incrementally increased between each run according to the scale factors specified in Table 1, and the sequence had continued until collapse, characterized by falling portions of the walls in the final runs. It should be noted that one extra induced-seismicity loading run (Run 10) had been included in the Flexible test. This extra loading run had been conducted between the tectonic runs, and had not been reported during the blind prediction competition.

**Table 1:** Loading sequence adopted for the testing of the specimens

	Loading Run		Stiff Specimen				Semi-Flexible Specimen				Flexible Specimen			
			Signal^i^	Scale^ii^	PTA^iii^		Signal	Scale	PTA		Signal	Scale	PTA	
	1		Induced	10%	0.04 g		Induced	10%	0.04 g		Induced	10%	0.05 g	
	2		Induced	20%	0.08 g		Induced	20%	0.08 g		Induced	20%	0.09 g	
	3		Induced	30%	0.13 g		Induced	30%	0.13 g		Induced	30%	0.13 g	
	4		Induced	50%	0.21 g		Induced	50%	0.21 g		Induced	50%	0.20 g	
	5		Induced	75%	0.32 g		Induced	75%	0.32 g		Induced	75%	0.28 g	
	6		Induced	100%	0.42 g		Induced	100%	0.42 g		Induced	100%	0.36 g	
	7		Tectonic	50%	0.27 g		Tectonic	50%	0.27 g		Tectonic	50%	0.28 g	
	8		Tectonic	75%	0.41 g		Tectonic	75%	0.41 g		Tectonic	75%	0.43 g	
	9		Tectonic	100%	0.55 g		Tectonic	100%	0.55 g		Tectonic	100%	0.57 g	
	10		Tectonic	125%	0.69 g		Tectonic	125%	0.69 g		Induced	100%	0.36 g	
	11		Tectonic	150%	0.82 g		Tectonic	150%	0.82 g		Tectonic	125%	0.70 g	
	12		Tectonic	175%	0.96 g		Tectonic	175%	0.96 g		Tectonic	150%	0.85 g	
	13		Tectonic	200%	1.10 g		Tectonic	200%	1.10 g		–	–	–	
	14		Tectonic	250%	1.38 g		–	–	–		–	–	–	
	15		Tectonic	300%	1.65 g		–	–	–		–	–	–	
	16		Tectonic	350%	1.93 g		–	–	–		–	–	–	

^i^ Type of seismic event from which the loading signal is extracted

^ii^ Applied to the 100%-scale signals shown in Fig. 2

^iii^ Peak Table Acceleration, recorded from the bottom shake table

### Review of the numerical models and changes made during post-diction

**Fig. 3:**
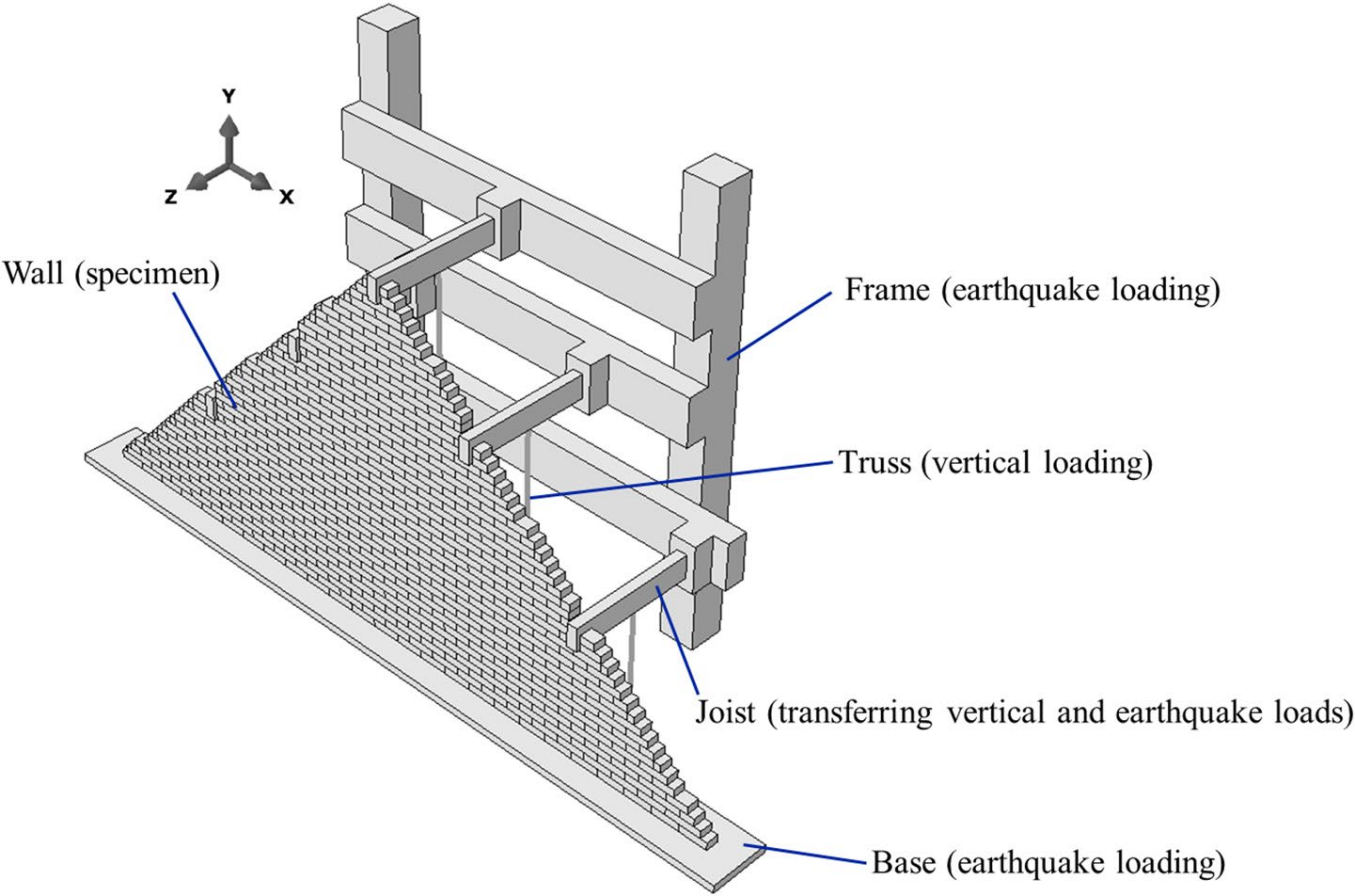
Overview of the numerical model produced during the ERIES SUPREME blind prediction competition

During the competition, the authors of this paper modeled two of the tested walls (those that were part of the competition), and their test set-up, as described in (Ghezelbash et al. 2026) and shown in Fig. 3. To optimize computational costs, some components, such as the shake table, top slab, and supporting columns, had been omitted. Other components, such as the loading frame and the joists, had been retained to accurately capture amplification effects in the transfer of dynamic motions to the walls. However, certain idealizations had been employed in the representation of these components to reduce computational demands. Specifically, the steel profiles of the loading frame had been replaced with solid rectangular members, maintaining only key dimensions such as length and height, spacing, and cross-sectional dimensions of the beams and columns. Moreover, the joists had been modeled as continuous rectangular prisms and had only been partitioned to distinguish between the mechanical properties of their steel and timber portions. The vertical loading system had also been explicitly modeled to account for variations in vertical loads caused by the movement and rotation of the joists. Yet, instead of simulating all its details, it had been simplified as a truss system. Despite these simplifications, the gable walls had been replicated in detail using expanded blocks and zero-thickness joints, following the numerical approach of (Ghezelbash et al. 2025; D’Altri et al. 2019; Ghezelbash et al. 2025), resulting in numerical wall specimens closely matching the experimental ones.

The modeling procedure has been detailed in (Ghezelbash et al. 2026) and consisted of several steps, such as generating geometries, simulating the masonry walls, connecting different components of the model, characterizing the mechanical properties of the numerical loading setup, implementing a Finite Element discretization scheme, prescribing boundary conditions and loading protocols, and setting up the dynamic solver. Herein, only the changes made during the post-diction phase to the numerical models compared to the reference models of the blind prediction (Ghezelbash et al. 2026) are presented. The changes included two minor adjustments based on the observations from prediction simulations and the experimental data published after the competition, as well as two primary re-calibrations aimed at enhancing the accuracy of the models.

The minor changes to improve the numerical models during the post-diction phase included: (1) assigning linear elastic behavior to all expanded blocks of the walls and (2) updating the input signals of dynamic loading runs. The implementation of the first change reflected findings from the prediction simulations, which had indicated no significant difference in the response of models with linear versus nonlinear blocks. Additionally, experimental evidence had not shown any notable damage to the bricks, suggesting that the collapse mechanisms of all three walls had been primarily governed by brick-to-mortar joints and purlin-wall connections. Additionally, elastic blocks had been found during the prediction phase to reduce numerical issues and improve computational efficiency. The implementation of the second change, prompted by the availability of more precise input motion data, replaced the reference signals used during blind prediction with accelerograms recorded during the actual tests. Consequently, the post-diction simulation of each wall employed the specific accelerograms obtained from the shake table and top slab during each loading run of the respective specimen. To ensure accuracy in reproducing the motions experienced by the gables during the tests, these accelerograms were processed using a band-pass Butterworth filter and linear baseline correction to remove noise. The same procedure used during the blind-prediction phase was adopted for processing the signals. First, approximately 0.3 s were cropped from the tail of each signal pair to ensure that, by the end of each loading run, the top and bottom boundaries of the numerical specimens returned to a similar OOP position. This step was taken to eliminate artificial residual OOP drift in the walls. Second, each signal was filtered using separately calibrated settings to ensure that the resulting displacement at the boundaries of the numerical specimens matched as closely as possible the displacement observed during the experiments.

The primary modifications made to the numerical models during the post-diction phase and after the aforementioned minor changes were: (1) a 25% increase in the friction coefficient used in the purlin-wall connections ($\:\mathrm{t}\mathrm{a}\mathrm{n}\:{{\upphi\:}}_{\mathrm{t}/\mathrm{m}}$) and (2) a 25% decrease in the tensile cohesive strength in the joints ($\:{\mathrm{f}}_{\mathrm{t}}$), combining the settings adopted in V6 and V3 variations of the prediction simulations (Ghezelbash et al. 2026), respectively. Based on insights from the parametric study conducted in the blind prediction phase, the $\:\mathrm{t}\mathrm{a}\mathrm{n}\:{{\upphi\:}}_{\mathrm{t}/\mathrm{m}}$ was identified as a critical parameter affecting the near-collapse response and collapse mechanism of the walls. Consequently, this coefficient was increased from the initial value of 0.6 to 0.75 in the post-diction analyses, still within the experimentally determined range reported in the literature (Almeida et al. 2020). In preliminary sensitivity studies, this change helped improve the transfer of dynamic motions to the walls. This effective transfer allowed more wall damage to appear before collapse while not excessively restricting sliding between the purlins and the wall. To compensate for the increased wall resistance resulting from the higher friction of purlin-wall connections, to allow easier cracking of the walls, and to ensure that the cracking initiation and collapse loading intensities (Peak Ground Accelerations, or PGAs) align with experimental results, the $\:{\mathrm{f}}_{\mathrm{t}}$ was reduced from the initial value of 0.18 MPa (the mean value from experimental recordings (Sharma et al. 2025)) to 0.14 MPa. This updated value was still within the 44% variation range observed in the material tests conducted during the ERIES SUPREME campaign (Sharma et al. 2025).

In addition to the aforementioned minor and primary changes during the post-diction phase, the possibility of improving the performance of the models by changing several other parameters was also pursued in a comprehensive sensitivity study that consisted of simulating 48 variations for each wall. However, those adjustments either resulted in worse agreement with the experimental outcomes or had no discernible impact on the response. For instance, data published after the blind prediction phase indicated that a vertical load of 4.5 kN had been applied at each purlin-wall connection, rather than the initially reported 4 kN. Incorporating this updated value into the numerical models led to unrealistic responses in the numerical setting, including the complete prevention of wall cracking and the occurrence of rigid body collapse (i.e., overturning) across all three specimens. The parametric study in the prediction phase had suggested the experimental loading apparatus may have exhibited lower stiffness than anticipated. Such lower stiffness may have resulted in reduced confinement of the walls than expected. This reduction may not have been recorded in the tests due to difficulties associated with the recording of the vertical load magnitude in experimental settings. Hence, the original 4 kN load was retained for the post-diction analyses. Other variations, including varying timber stiffness to 50% lower and higher, using a different Finite Element type (with reduced integration points or a tetrahedral shape), increasing the number of contact points in the thickness direction of the purlin-wall interface, and adopting different damping ratios, also did not lead to increased agreement with the experimental outcomes. Consequently, it was decided to exclude these variations from the post-diction phase and confine the changes to using elastic expanded blocks and updated dynamic loading input signals, increasing the friction between purlins and walls, and decreasing the tensile strength of the zero-thickness joints. This decision also aligned with the internal goal of maintaining a straightforward modeling methodology without introducing intricate fine-tuning adjustments. Indeed, the adjustment of $\:{\mathrm{f}}_{\mathrm{t}}$ and $\:\mathrm{t}\mathrm{a}\mathrm{n}\:{{\upphi\:}}_{\mathrm{t}/\mathrm{m}}$ already significantly improved the simulations and provided the best fit with the experimental outcomes across all variations without the need for further calibrations.

## Post-diction numerical simulation results

**Fig. 4:**
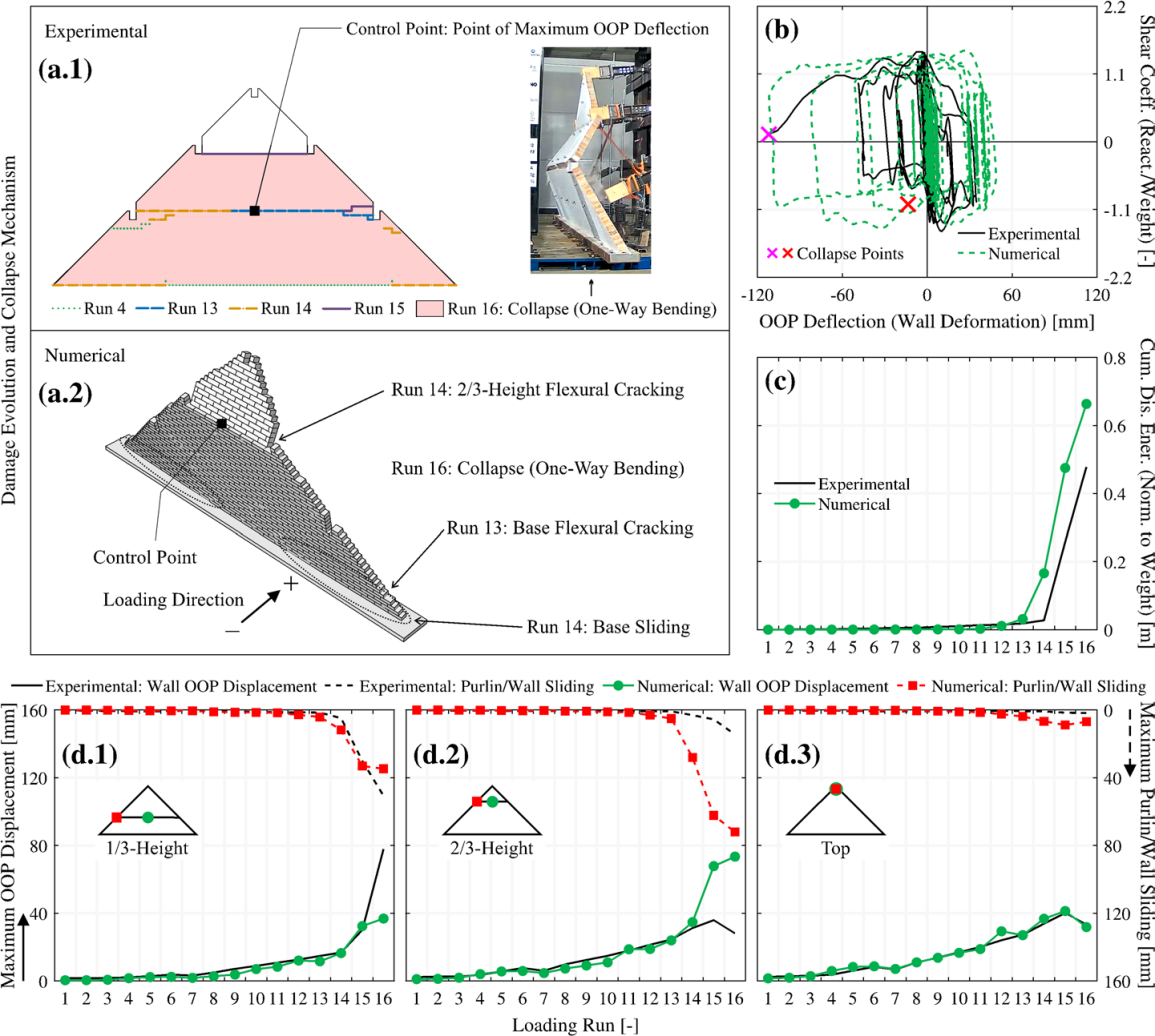
Results of post-diction simulation for the Stiff test: experimental (**a.1**) and numerical (**a.2**) crack patterns and collapse mechanisms, hysteretic response (**b**), cumulative energy dissipation (**c**), and maximum OOP displacement and purlin/wall sliding recorded at 1/3-height (**d.1**), 2/3-height (**d.2**), and top (**d.3**) elevations of the wall during each loading run. Experimental and numerical reaction forces and energy dissipations have been normalized by the weight of each experimental wall (17.5 kN) and the total weight of each numerical wall and loading set-up (44 kN), respectively

**Fig. 5:**
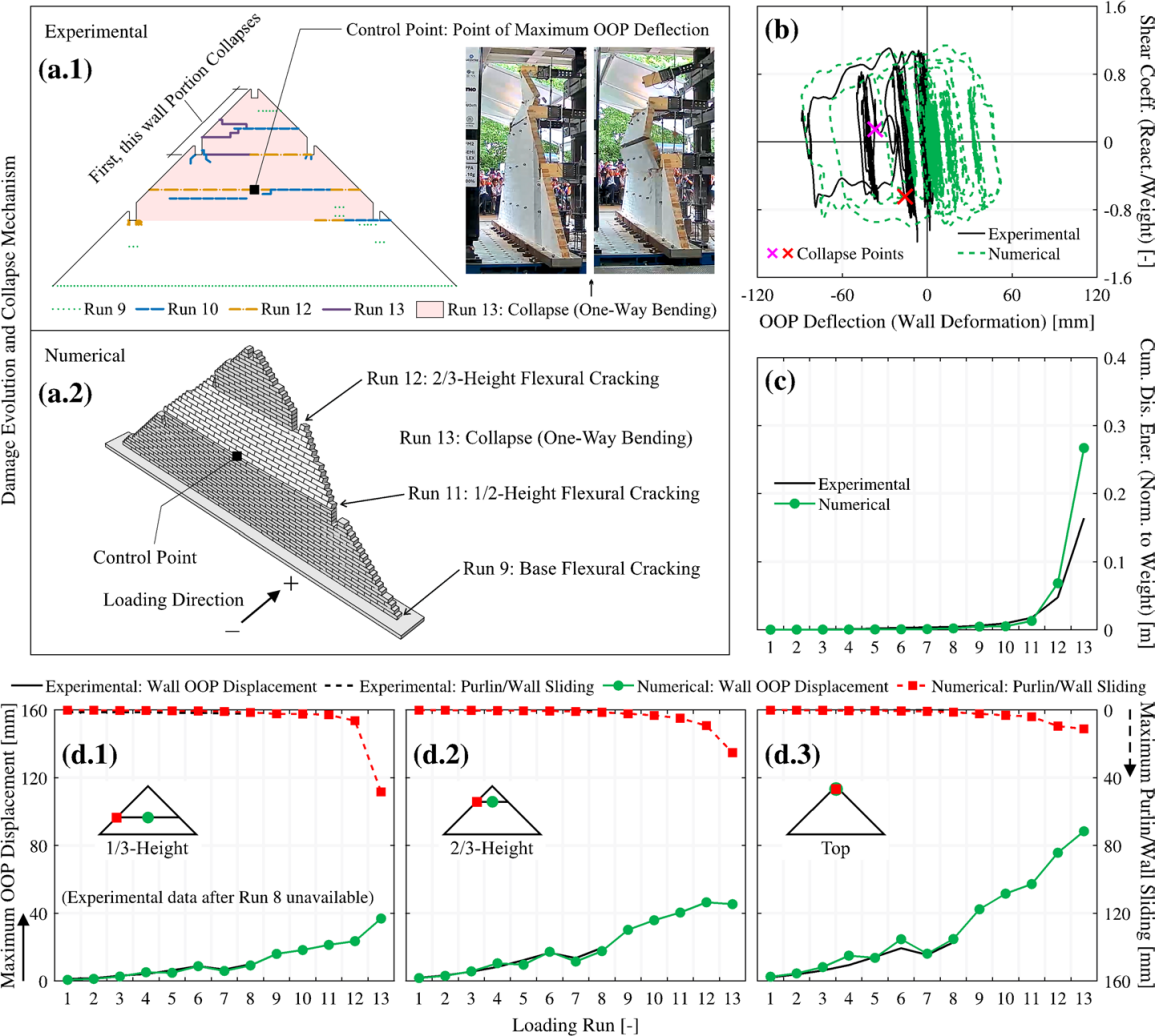
Results of post-diction simulation for the Semi-Flexible test: experimental (**a.1**) and numerical (**a.2**) crack patterns and collapse mechanisms, hysteretic response (**b**), cumulative energy dissipation (**c**), and maximum OOP displacement and purlin/wall sliding recorded at 1/3-height (**d.1**), 2/3-height (**d.2**), and top (**d.3**) elevations of the wall during each loading run. Experimental and numerical reaction forces and energy dissipations have been normalized by the weight of each experimental wall (17.5 kN) and the total weight of each numerical wall and loading set-up (44 kN), respectively

**Fig. 6:**
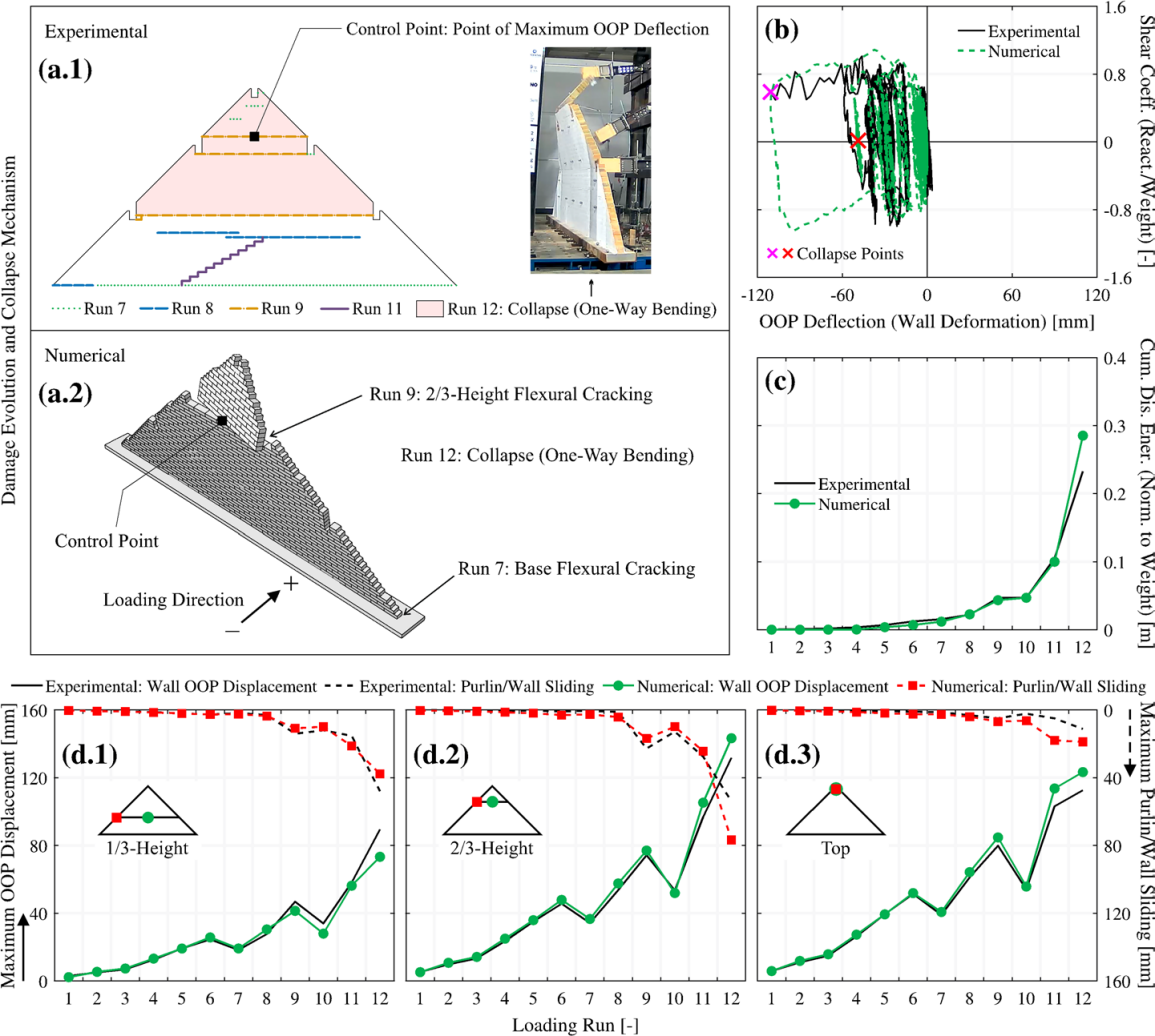
Results of post-diction simulation for the Flexible test: experimental (**a.1**) and numerical (**a.2**) crack patterns and collapse mechanisms, hysteretic response (**b**), cumulative energy dissipation (**c**), and maximum OOP displacement and purlin/wall sliding recorded at 1/3-height (**d.1**), 2/3-height (**d.2**), and top (**d.3**) elevations of the wall during each loading run. Experimental and numerical reaction forces and energy dissipations have been normalized by the weight of each experimental wall (17.5 kN) and the total weight of each numerical wall and loading set-up (44 kN), respectively

The results of post-diction simulations of the Stiff, Semi-Flexible, and Flexible specimens have been presented in Figs. 4, 5, and 6, respectively. Damage evolution and failure mechanisms, hysteretic force-displacement response and energy dissipation, and the maximum OOP displacement and purlin-wall sliding recorded during each dynamic loading run have been included in the figures. The numerical deformed shapes (Fig. 4.a.1, 5.a.1, and 6.a.1), ×10 magnified, were captured at the moment of maximum deformation during the final loading run. Comparison of the numerical results with the experimental outcomes showed overall good agreement, indicating significantly better agreement with the tests compared to the best-fitting prediction simulations (V1 models in (Ghezelbash et al. 2026)). The numerical models for the three specimens have been developed consistently during post-diction, maintaining the same geometrical, material, and damping properties, as well as identical dynamic analysis procedures and solver settings across the three specimens. The sole difference between the three models lies in the dynamic loading sequence and signals specific to each specimen. More detailed discussion of the remaining slight discrepancies between the numerical and experimental results has been provided in the following paragraphs.

Regarding damage patterns and collapse mechanisms, the numerical models showed a cracking sequence consistent with the experimental observations in terms of crack locations and the loading intensities at which they form. The primary difference was the absence of the horizontal crack observed at the 1/3-height elevation in the experimental walls from the numerical models. For instance, while the experimental Stiff wall exhibited a one-way bending failure mechanism with a crack at the 1/3-height elevation (Fig. 4.a.1), the numerical model instead showed a crack at the 2/3-height elevation (Fig. 4.a.2). Similarly, while the bottom portion of the experimental Semi-Flexible and Flexible walls remained stable during collapse (Figs. 5.a.1 and 6.a.1, respectively), their numerical counterparts (Fig. 5.a.2 and Fig. 6.a.2, respectively) exhibited one-way bending mechanisms involving the entire wall, with the bottom portion of the walls bending with parts on top of them. This discrepancy may have stemmed from the bottom purlins in the experimental setup transmitting different vertical loads than the assumed 4.0 kN or exhibiting greater frictional resistance, thus enhancing the stability of the bottom portions.

Regarding the hysteretic response (Fig. 4.b, Fig. 5.b and Fig. 6.b), the numerical models (dashed green curves) showed strong agreement with the experimental data (solid black curves) in terms of peak OOP resistance, peak deflections, and the shape of hysteretic cycles. However, discrepancies were observed in energy dissipation (Fig. 4.c, Fig. 5.c and Fig. 6.c), with numerical models exhibiting up to 50% greater total energy dissipation than the experiments. Although the shape of the numerical and experimental hysteretic cycles generally matched for the three walls, the numerical models demonstrated up to two additional full cycles of deformation before collapse, likely due to the assumed constant 5% Rayleigh damping ratio ($\:{{\upzeta\:}}_{\mathrm{R}}$) differing from the actual dissipation mechanisms of the tested walls. Although prediction simulations had also shown such influence of the damping ratio on energy dissipation and hysteretic cycles, further fine-tuning of the damping ratio did not improve the accuracy of the results. Moreover, given that the global numerical hysteretic behavior adequately reflected the experimental response, additional adjustments were deemed unnecessary.

In the hysteretic plots, the deflection data of each wall has been obtained from control points specific to its collapse mechanism. These deflections are different from total displacements and have been calculated as pure deformations of the walls, i.e., OOP displacements of the control points minus the OOP displacement of the loading frame. In the Flexible wall, both numerical and experimental deflections were recorded at a point at the mid-length and 2/3-height elevation of the wall. For the Semi-Flexible wall, the data were collected at a control point at the 1/2-height elevation. Meanwhile, for the Stiff wall, the experimental control point was located at the 1/3-height elevation, while the numerical control point was assumed at the 2/3-height, reflecting the difference in the location of the peak deflections between the numerical model and the experiment. The calculation of the shear coefficient and energy dissipation followed the methodology used in the prediction phase (Ghezelbash et al. 2026).

In the benchmark experiments (Sharma et al. 2025), collapse was identified as the inability of the wall to return to equilibrium conditions, i.e., the physical falling of some or all of its portions (Adhikari and D’Ayala 2020). In the numerical simulations of this study, although the discontinuum modeling approach can represent such phenomena, the full progression of collapse (i.e., complete detachment and fall of loosened wall portions) could not always be captured due to the limitations associated with the use of an implicit solver. Hence, the onset of collapse has been adopted as the indicator of numerical collapse. The large deformations observed at numerical control points (exceeding 80% of wall thickness and surpassing thresholds reported in (Melatti and D’Ayala 2021; Vaculik and Griffith 2017; Mercuri et al. 2020; Walsh et al. 2018; Casapulla et al. 2019) for walls with similar failure mechanisms), along with significant sliding between purlins and walls (leading to detachment), in the final stages of the analyses were indicative of the collapse with the adopted definition and the appropriateness of using collapse onset as a representative of collapse. According to this convention, the last moment simulated in the analyses was selected as the numerical collapse point, disregarding the full progression of collapse.

The recorded maximum OOP displacements (green solid curves with square markers) and purlin/wall slidings (red dashed curves with circle markers) in different dynamic loading runs of the numerical models aligned closely with the experimental data (solid and dashed black curves, respectively), showing substantial improvements over the prediction phase results (Fig. 4.d, Fig. 5.d and Fig. 6.d). Although for the Semi-Flexible wall, data for loading runs beyond Run 8 were not available as the corresponding sensors had been removed during the test (Sharma et al. 2025). The Flexible wall model showed excellent agreement with the experimental results, while the Stiff wall exhibited a minor mismatch due to the higher placement of the crack in the numerical simulation, resulting in large OOP displacements and sliding at the 2/3-height rather than the 1/3-height observed experimentally. Despite this mismatch, the maximum OOP displacements and sliding at the 2/3-height elevation of the numerical Stiff wall still aligned with the data obtained at the 1/3-height elevation of the experimental wall, indicating that although the location of the crack was changed in the numerical model, the experimentally observed deformation and sliding capacities were effectively maintained. In the figures, the maximum purlin/wall sliding curves have been presented in a reversed direction within the plots to distinguish them from the maximum OOP displacement curves, starting from zero at the top edge and increasing downward.

## Sensitivity study of unexplored structural configurations

Sensitivity studies conducted during the blind prediction phase, and previous research using the numerical modeling approach of this study (Ghezelbash et al. 2025; Ghezelbash et al. 2025), have demonstrated its capability to complement experimental investigations in studying unexplored structural configurations, especially when physical testing is limited by time, budget, or facility constraints. Additionally, the post-diction numerical vs. experimental comparisons presented in Sect.  3 highlighted the reliability of the modeling approach in accurately replicating the real-world behavior of gable walls under various loading conditions. Therefore, the potential of using numerical models to expand the current understanding beyond the experimental findings from the ERIES SUPREME gable wall tests (Sharma et al. 2025) has been examined in this section. Accordingly, the numerical models have been extended to the simulation of wall configurations that, although not considered in the original tests, represent structural configurations that can be found in the field.

### Numerical model variations

The additional structural configurations considered have been outlined in this section. Since the influence of modeling parameters had been thoroughly investigated during the prediction phase, the focus was placed on examining the effects of structural variations in the models, specifically involving different purlin-wall connection types and pre-existing damage, on the OOP resistance and failure mechanism of the gables. These scenarios reflected real-world conditions of gables, and were prioritized due to their practical relevance: they addressed critical factors that can significantly alter the structural response of gable walls under seismic loading. Specifically, the seven numerical variations listed in Table 2 were developed in collaboration with the organizers of the ERIES SUPREME campaign (Sharma et al. 2025), with each variation designated as “Vi,” and the V0 models being the recalibrated post-diction models (described in Sect.  2.3 and 3) and serving as the reference configurations.

**Fig. 7:**
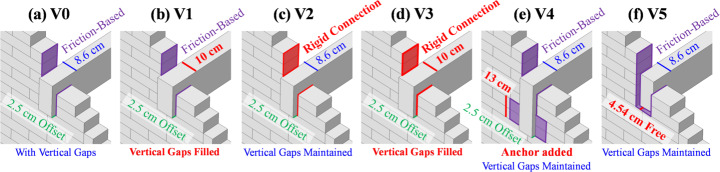
Different purlin-wall connections adopted in the numerical model variations of the sensitivity study: the original set-up in the reference V0 models (**a**), the no-gap connection in V1 models (**b**), the rigid-contact connection in V2 models (**c**), the combined no-gap rigid-contact connection in V3 models (**d**), the anchored connection adopted in V4 models (**e**), and the reduced-contact configuration of V5 models (**f**)

Variations V1 to V5 were conceived with different purlin-wall connection configurations as illustrated in Fig. 7, where the differences between each variation and the V0 models are highlighted with red color and bold text. The V1 models were designed with no vertical gaps between the sides of the purlins and their surrounding bricks, to examine whether such construction scenarios result in a more two-way bending OOP response. In these models, friction-based connections, similar to V0 models, were retained at both vertical and horizontal sides of the wedges. The filling of vertical gaps was achieved by enlarging the purlin cross-section, extending it to cover the entire 10 cm wedge space. In V2 models, the original purlin geometries were maintained, yet the friction-based connections were replaced with fully rigid ones to simulate scenarios with strong wall-roof connections. This was achieved by adopting purely elastic contacts with normal and shear stiffness values equal to those used for the zero-thickness joints ($\:{\mathrm{k}}_{\mathrm{n}\mathrm{t}}$=241.4 N/mm^3^ and $\:{\mathrm{k}}_{\mathrm{s}}$=103.1 N/mm^3^ in (Ghezelbash et al. 2026)). The settings of V1 and V2 variations were combined in the V3 models, by featuring expanded purlins with no vertical gaps connected to the walls via rigid connections. In V4 models, retaining the friction-based connections of the V0 model and the vertical gaps at the sides of the purlins, a notch was introduced at the bottom of each purlin on the side of the wall facing away from the loading frame. The notch, designed to resemble conventional anchoring systems (Tomassetti et al. 2019), has a height of 13 cm, extending to two brick rows below the wedges, and was connected to the wall surface via the same friction-based contact used at other regions of the purlin-wall connections. In this configuration, asymmetric resistance was imposed: the walls were restrained when sliding away from the purlins (in the positive z-direction in Fig. 1) but remained unrestricted when they slid in the opposite direction (negative z-direction in Fig. 1). In the V5 models, the original friction-based connections and purlin geometries were maintained, but the purlin-wall contact surfaces beneath purlins were reduced to 80% of the wall thickness. This reduction was done moving the walls (and their underlying base) away from the loading frame by 7.04 cm. This variation simulated walls dislocated from their original support positions by deformations induced during past earthquake events.

Variations V6 and V7 were developed to examine the impact of pre-damage on the OOP response of the gables, each following a different methodology. In V6 models, pre-damage was introduced directly by deforming (and, hence, damaging) the walls before the dynamic loading runs. Conversely, an indirect approach was adopted in V7 models, where pre-damage effects were replicated by a priori weakening the joints at specific regions of the walls. Specifically, the walls in V6 models were subjected to a full cycle of 3.74 cm OOP displacement (equivalent to 1.2% of the loading frame height) applied at the top of the loading frame after gravitational and vertical loading and before the dynamic runs. The displacement magnitude was calibrated in such a way that the largest crack width caused by it in the walls reaches twice the length of the softening regime in the zero-thickness joints ($\:2{\mathrm{u}}_{\mathrm{k}}=1.2\:\mathrm{m}\mathrm{m}$). This resulted in complete tensile softening and the loss of shear ($\:\mathrm{c}$) and tensile ($\:{\mathrm{f}}_{\mathrm{t}}$) strengths in the entire wall thickness at the crack location. The cyclic OOP deformation consisted of three stages: the frame was first pushed toward the wall by 3.74 cm, then pulled away to the same drift magnitude in the reverse direction, and finally returned to the zero-displacement position. In the numerical models of this study, this loading sequence generated a crack at the base of the walls, leaving only frictional resistance between the walls and the foundations. To investigate the possibility of mimicking the pre-damage effects observed in the V6 models while avoiding the computational demands and the complexities of directly applying pre-deformations, V7 models were developed in which, instead of the deformation sequence utilized in V6 models, the cohesive shear ($\:\mathrm{c}$) and tensile strengths ($\:{\mathrm{f}}_{\mathrm{t}}$) of the zero-thickness joints at the base of the walls were manually reduced to 0 MPa.

Due to the changes in the modal response of the V1 to V5 models as a result of altering their geometries, their Rayleigh damping parameters were re-calibrated with the new frequencies of their 1st and 4th natural deformation modes (the same modes considered for the blind prediction models). Additionally, to account for the possibility of some numerical variations exhibiting higher resistance compared to the V0 models, an extended dynamic loading sequence was implemented, wherein additional loading runs were introduced to the original sequences listed in Table 2. In these additional runs, placed after the final loading runs of the original sequences, the 100%-intensity tectonic loading signals from loading run 9 in each test were adopted and were scaled to intensities incrementally increased by 25% in each subsequent run. For instance, in the added runs 17, 13, and 14 for the Stiff, Semi-Flexible, and Flexible specimens, respectively, the tectonic signal from loading run 9 of the same tests but with scale factors of 325%, 225%, and 175%, respectively, were used (25% higher than the scale factors 350%, 200%, 150%, in their previous loading runs, respectively).

**Table 2:** Different numerical variations considered for the extrapolation of the findings

	Variation		Masonry Properties				Purlin-Wall Connection				Loading Assumptions				
			Blocks		Joints $\:{\mathrm{f}}_{\mathrm{t}}$		Geometry		Behavior		Loading Signals		Wall Pre-Damage		
	V0		Linear		0.135 MPa		Original		$\:\mathrm{t}\mathrm{a}\mathrm{n}\:{{\upphi\:}}_{\mathrm{t}/\mathrm{m}}=0.75$		Per-Run Test Records		None		
	V1		Linear		0.135 MPa		**Gaps Filled** ^**i**^		$\:\mathrm{t}\mathrm{a}\mathrm{n}\:{{\upphi\:}}_{\mathrm{t}/\mathrm{m}}=0.75$		Per-Run Test Records		None		
	V2		Linear		0.135 MPa		Original		**Rigid** ^**ii**^		Per-Run Test Records		None		
	V3		Linear		0.135 MPa		**Gaps Filled**		**Rigid**		Per-Run Test Records		None		
	V4		Linear		0.135 MPa		**Anchored** ^**iii**^		$\:\mathrm{t}\mathrm{a}\mathrm{n}\:{{\upphi\:}}_{\mathrm{t}/\mathrm{m}}=0.75$		Per-Run Test Records		None		
	V5		Linear		0.135 MPa		**Reduced Contact** ^**iv**^		$\:\mathrm{t}\mathrm{a}\mathrm{n}\:{{\upphi\:}}_{\mathrm{t}/\mathrm{m}}=0.75$		Per-Run Test Records		None		
	V6		Linear		0.135 MPa		Original		$\:\mathrm{t}\mathrm{a}\mathrm{n}\:{{\upphi\:}}_{\mathrm{t}/\mathrm{m}}=0.75$		Per-Run Test Records		**via Deformation** ^**v**^		
	V7		Linear		0.135 MPa		Original		$\:\mathrm{t}\mathrm{a}\mathrm{n}\:{{\upphi\:}}_{\mathrm{t}/\mathrm{m}}=0.75$		Per-Run Test Records		**via Material Input** ^**vi**^		

^i^ The cross-section of the purlins is enlarged in width to fill the entire 10 cm of the wedges

^ii^ Elastic contacts with large normal and transversal stiffnesses equal to those of the mortar joints are used

^iii^ A notch is added to the bottom of purlins on the side of the walls facing away from the loading frame

^iv^ The walls are moved away from the loading frame by 7.04 cm, so the purlins sit only on 80% of wall thickness

^v^ OOP cyclic drift applied at the top of the loading frame, causing a maximum crack width of 1.2 mm at the base of the walls

^vi^ Tensile and shear cohesive strengths of the base joint (connecting the walls to the base) are manually reduced to 0 MPa

### Analysis procedure

**Fig. 8:**
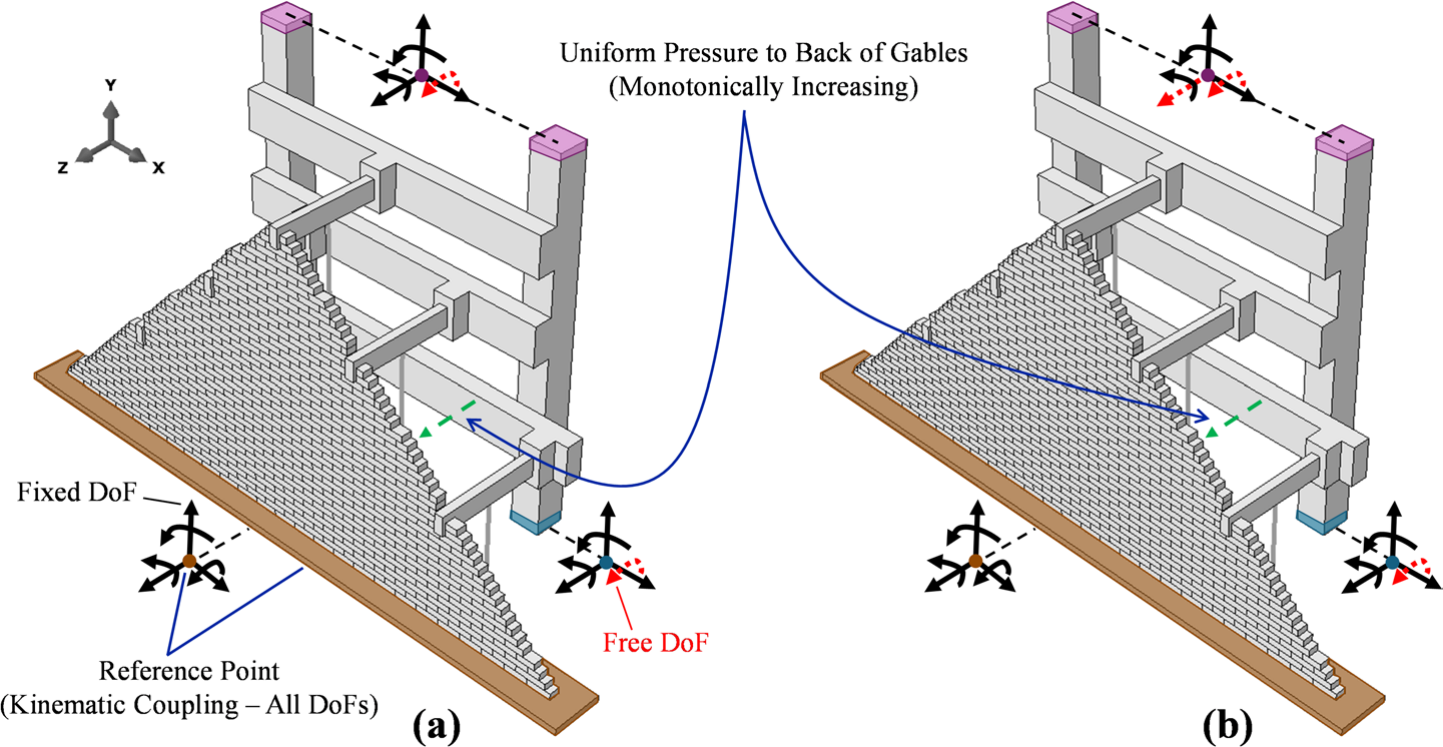
Boundary conditions and loading procedure used for the pushover analyses in the sensitivity study: Fixed-Frame scenario with no frame-top OOP movement (**a**), and Free-Frame scenario with free frame-top OOP movement. The reference points are located at the same positions used in the prediction analyses described in (Ghezelbash et al. 2026). For visualization clarity, they have been virtually repositioned in the figure

In addition to the dynamic analysis of all numerical variations of each wall, static pushover analyses were conducted to gain a more comprehensive understanding of the effects associated with the considered structural configurations. The pushover simulations were organized into two scenarios, each employing distinct boundary conditions as illustrated in Fig. 8. In the first scenario (referred to as the “Fixed-Frame” case and shown in Fig. 8.a), boundary conditions analogous to the Rigid Case (representing the interaction of gables with rigid diaphragms) were used. Namely, the OOP translation at all boundaries (the base and bottom of the trusses, as well as the base and top of the loading frame) was constrained. A uniform pressure was applied to the wall surface facing the loading frame, pushing the wall away from the frame, i.e., in the positive direction of the z-axis. The pressure was increased monotonically until structural collapse, defined as cracking and falling of the entire or portions of the wall, occurred. The same limitation of the implicit solver in tracing the full progression of collapse described for the post-diction analyses was encountered here. However, the adopted procedure is deemed valid, as the simulations captured the response up to very large displacements and wall-purlin separations, which are indicative of physical collapse (Melatti and D’Ayala 2021). In the second scenario (referred to as the “Free-Frame” case and shown in Fig. 8.b), the same loading protocol and constraints were applied as in the first scenario, with the exception that the OOP translation at the top of the loading frame was released, allowing it to freely displace in the positive z-direction during the application of surface pressure. This second scenario was intended to simulate an extreme scenario in which any contribution from roof stiffness to the confinement and OOP resistance of the wall was entirely neglected. To complement both the dynamic and pushover analyses, modal analyses were conducted on the pristine models to extract the frequencies associated with their first fundamental deformation mode. These modal analyses were performed following the approach used during the prediction phase (Ghezelbash et al. 2026), i.e., under the same boundary conditions as those in the Fixed-Frame pushover scenario (Fig. 8.a) and prior to the application of surface pressure.

### Simulation results

**Fig. 9:**
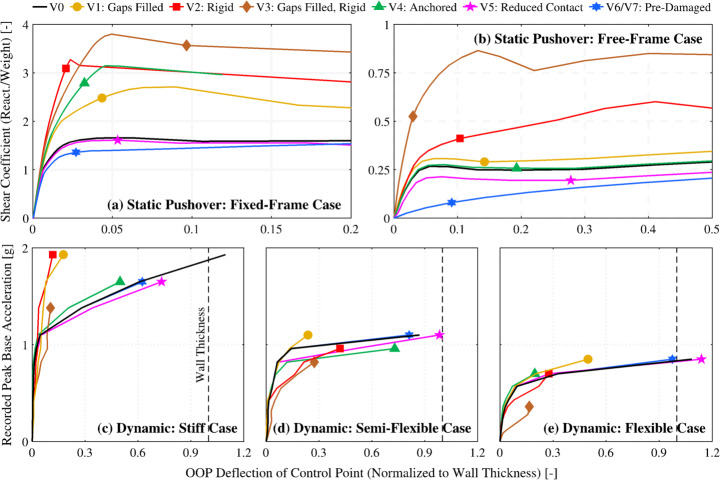
Static pushover force-displacement response and dynamic IDA curves of different numerical variations under different loading scenarios

**Fig. 10:**
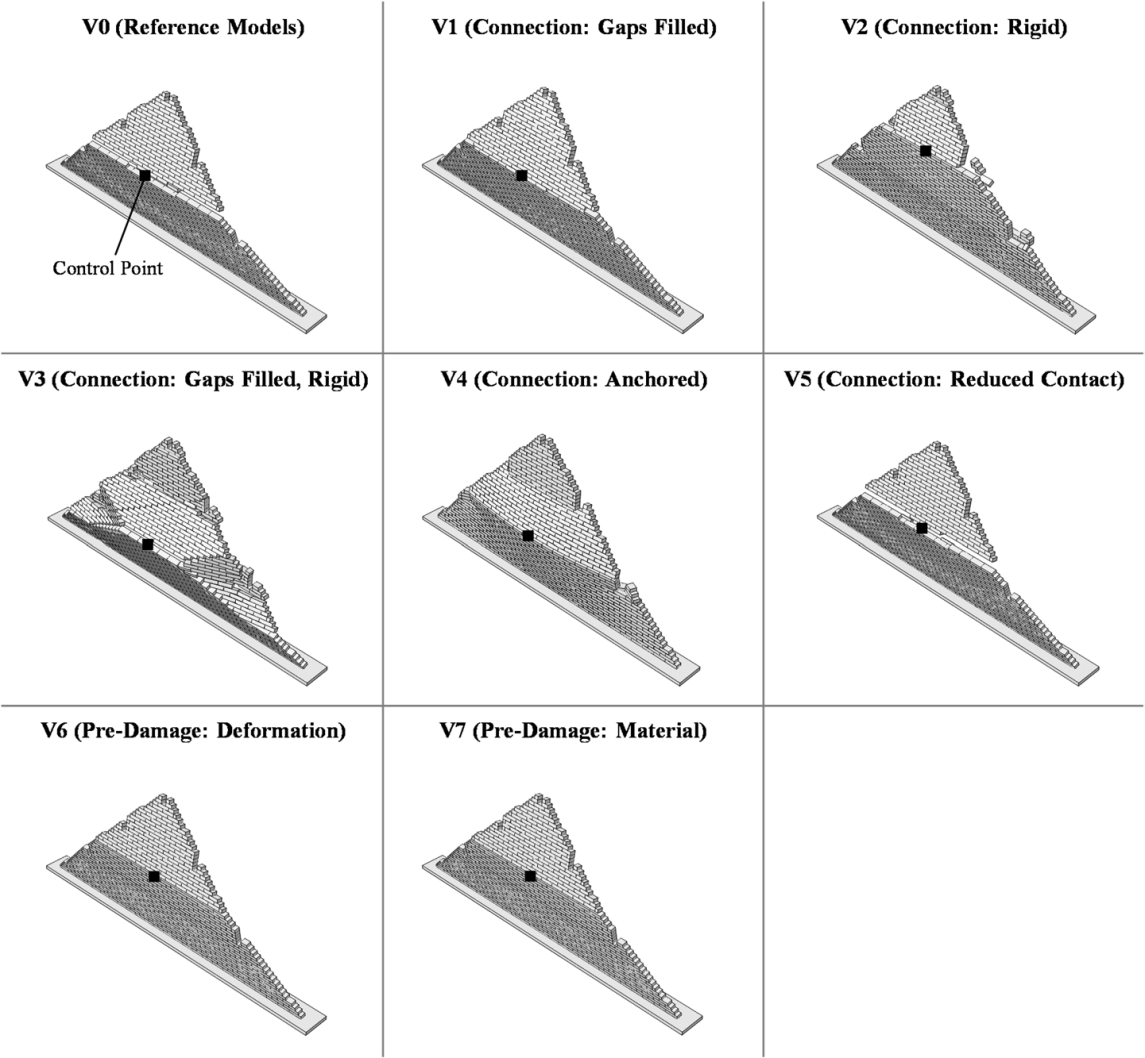
Failure mechanisms observed across different numerical model variations of the sensitivity study under fixed-frame static pushover loading

**Fig. 11:**
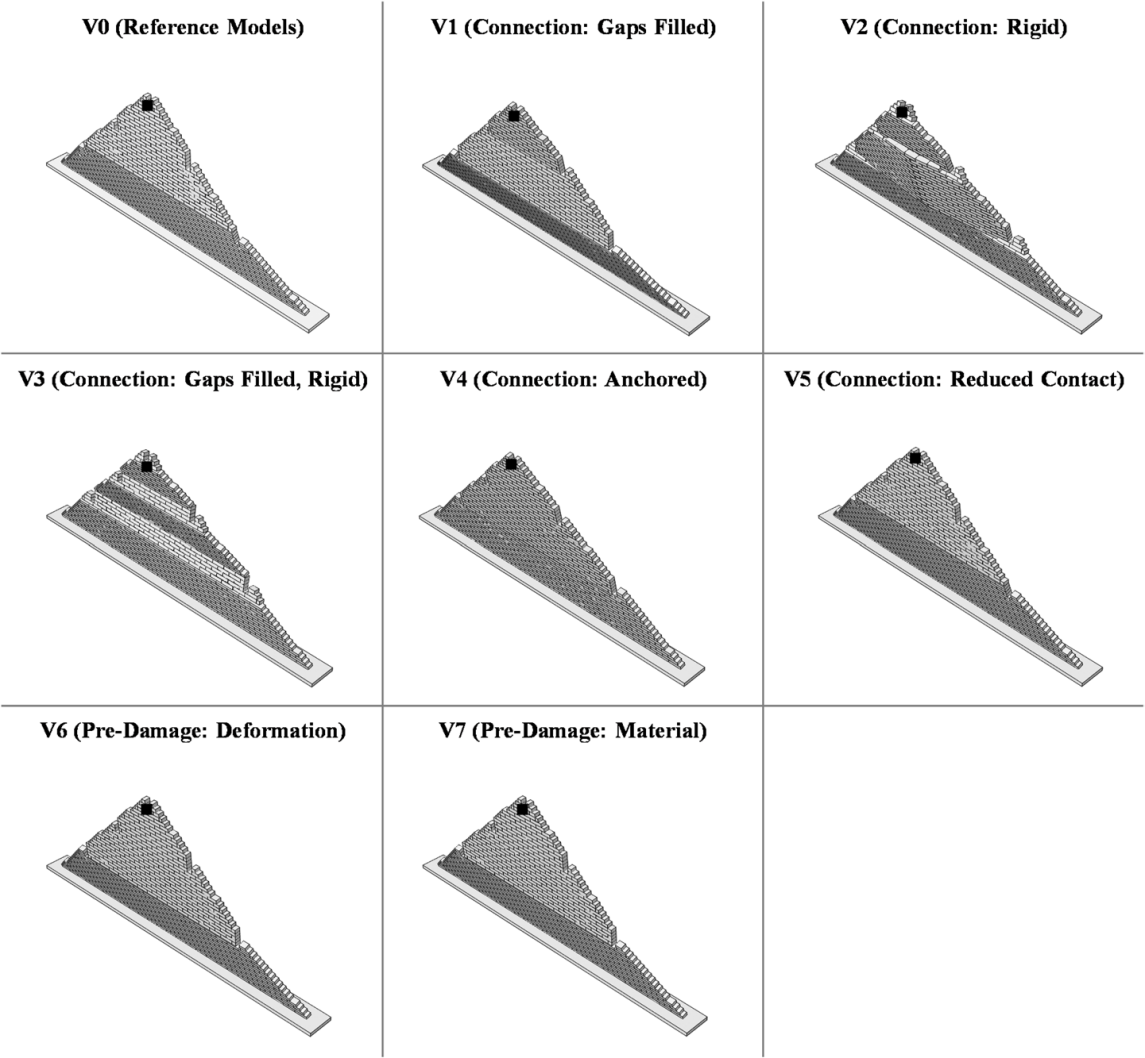
Failure mechanisms observed across different numerical model variations of the sensitivity study under free-frame static pushover loading

**Fig. 12:**
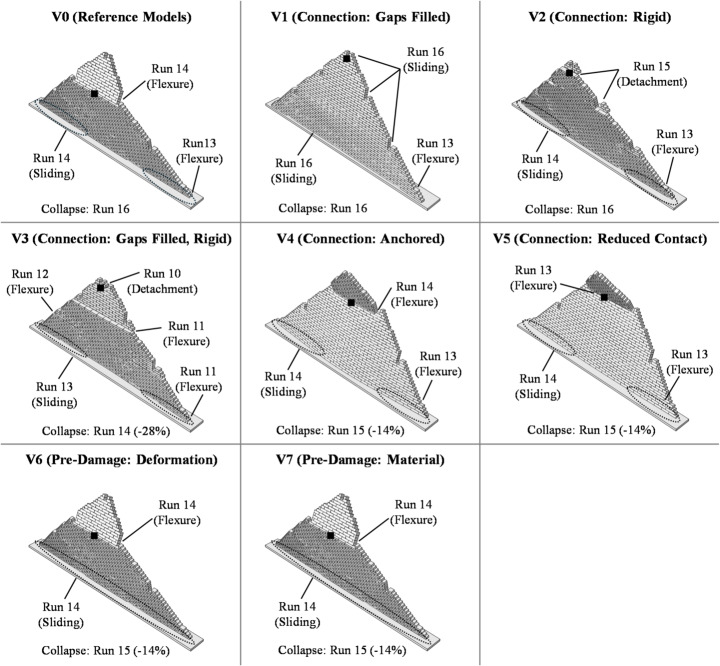
Failure mechanisms observed across different numerical model variations of the sensitivity study under dynamic loading history of the stiff case

**Fig. 13:**
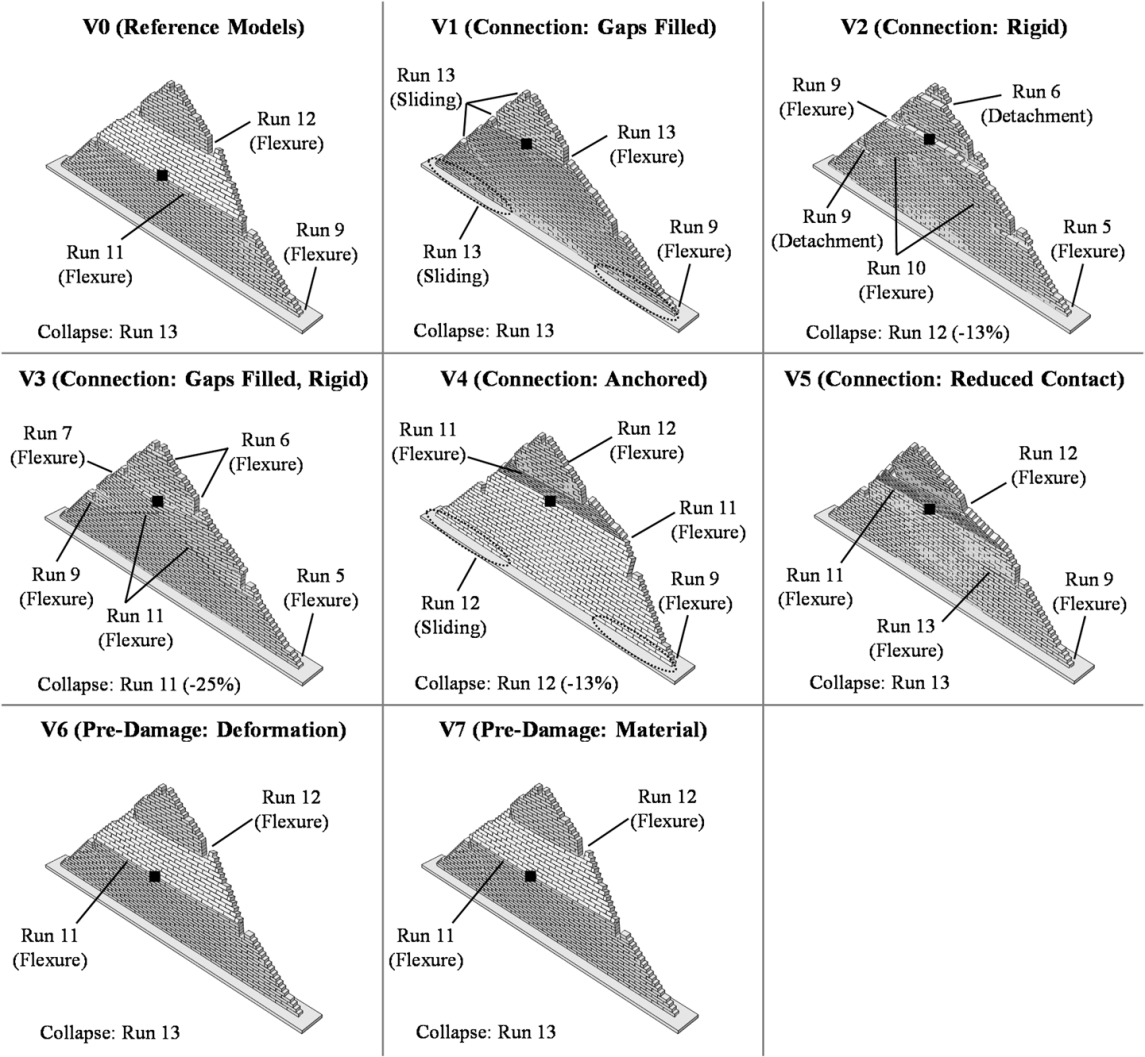
Failure mechanisms observed across different numerical model variations of the sensitivity study under dynamic loading history of the semi-flexible case

**Fig. 14:**
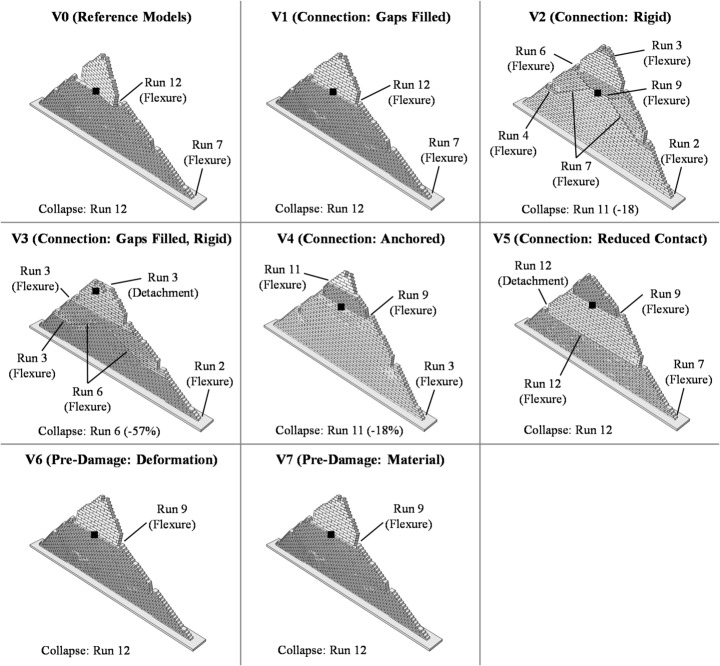
Failure mechanisms observed across different numerical model variations of the sensitivity study under dynamic loading history of the flexible case

The results of the simulations performed during the sensitivity study have been presented in this section. In Fig. 9, the static force-displacement responses and dynamic IDA curves of all variations under pushover and dynamic loading, i.e., the applied PBA versus the maximum OOP displacement observed in each loading run, have been shown. The crack evolution and collapse mechanisms have been shown in Figs. 10, 11, 12, 13, and Fig. 14. The deformed shape of each specimen has been ×10 magnified for clearer illustration of the cracks. Pushover reaction forces have been shown as a coefficient of the weight of the walls (17.5 kN each) to facilitate comparison with the loading PBAs in the dynamic simulations. The control points used to obtain the maximum OOP deflection in each simulation have been shown in Figs. 10, 11, 12, 13 and 14 and were selected according to the observed crack pattern. The maximum OOP displacements were calculated as the maximum deformation of the control point with respect to the loading frame, i.e., displacements solely due to internal OOP deformations of the wall. In Fig. 9, the curves of the reference V0 variations have been shown with solid black lines, while the curves of other variations have been shown with colored lines and were highlighted with different markers. Finally, varying y-axis scales have been employed between Fig. 9.a and Fig. 9.b for the clear visualization of the pushover curves, whereas a consistent y-axis scale has been maintained across Fig. 9.c, Fig. 9.d, and Fig. 9.e showing dynamic IDA curves.

**Fig. 15:**
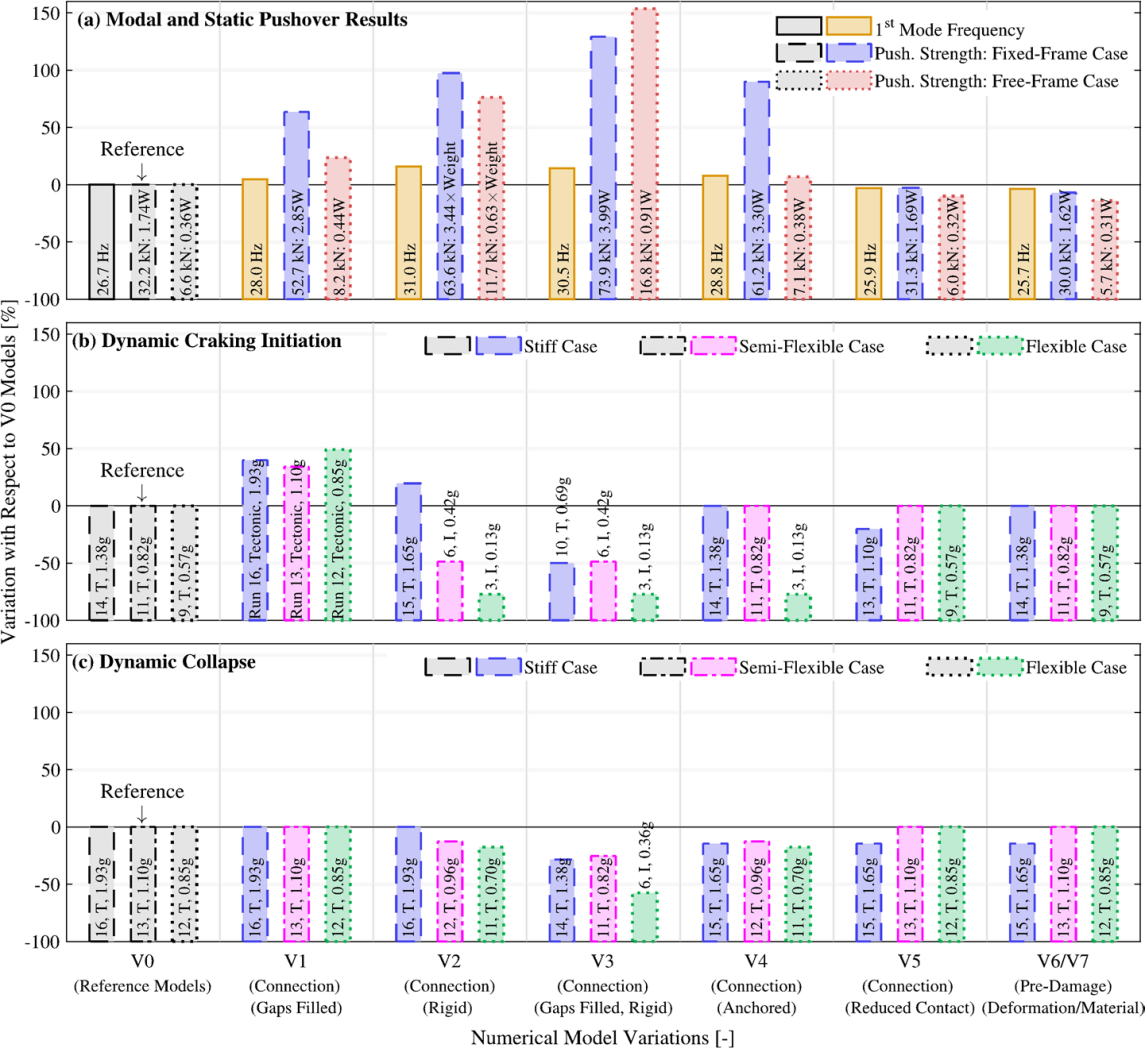
Summary of the results of the modal, static pushover, and dynamic analysis of different numerical model variations

The first-mode frequencies, OOP pushover strengths, and Peak Base Accelerations (PBAs) corresponding to the initiation of cracking and collapse of the models during dynamic simulations have been summarized in Fig. 15. The bars of the V1 to V7 models have been normalized to the corresponding value of their data in the V0 reference variations. The dynamic cracking initiation was identified as the loading run in which a crack with a maximum opening exceeding 0.3 mm appears in any region of the walls except for their base. The 0.3 mm threshold corresponds to the passing of half the softening branch ($\:{\varDelta\:}_{\mathrm{k}}$=$\:{{\updelta\:}}_{\mathrm{k}}$=0.6 mm) in the joint, which corresponds to loss of 50% of its cohesive tensile and shear strength. The exclusion of base cracks was done because such damage typically exists regardless of the level of damage, and, as explained in Sect.  4.4, has a minimal effect on the structural response. In the histograms of Fig. 15.a, first-mode frequencies have been shown with yellow bars with solid lines, pushover strengths of the Fixed-Frame case with blue bars with dashed lines, and Free-Frame pushover strengths with red bars with dotted lines. Pushover OOP strengths are shown as a coefficient of the weight of the walls (18.5 kN, indicated with “W”). In Fig. 15.b and Fig. 15.c, the dynamic cracking and collapse PBAs of the Stiff and Flexible walls have been shown via blue bars with dashed lines and green bars with dotted lines, respectively, while the data of the Semi-Flexible wall are shown with pink bars with dashed-dotted lines. The illustration of the outputs of V1-7 variations has been normalized to their corresponding reference values in the V0 models, shown with gray bars. A detailed discussion of the outcomes has been brought in the following. Owing to their alignment with the dynamic response of the Stiff V0 model (explained in Sect.  4.4), the Fixed-Frame pushover analyses have been presented using the same color and bar style as the dynamic Stiff case results in Fig. 15. The Free-Frame static results have been depicted using the bar style of the dynamic Flexible case, yet a different color has been used to reflect their deviation from the response observed in the Flexible V0 model.

Collapse in the dynamic simulations was again identified through large displacements indicative of the onset of physical instability and wall overturning (Melatti and D’Ayala 2021), consistent with the V0 simulations. However, in certain variations, such as the V1-3 Stiff models, collapse as physical instability occurred before such large displacements. In these cases, collapse was primarily triggered by localized damage at the purlin-wall connections or loss of contact at the wall base. These conditions prevented stabilization of the walls and hindered the transfer of additional dynamic loads to the walls. Consequently, numerical instability was encountered due to the use of the implicit solver, halting the progression of the simulation. As such, the final analysis step was taken as the point of collapse. This limitation is not expected to affect the validity of the results: observations of purlin-wall or floor-wall detachment are considered in real-world practice as indicators of collapse onset or near-collapse conditions, in buildings with gable walls. A relevant example can be found in (Graziotti et al. 2017), where a building-level dynamic test had been terminated when extensive cracking was observed between the gable wall and the supporting floor, as well as at the purlin-wall connections. Despite the rest of the building retaining stability, the specimen had been assumed to have reached near collapse conditions upon showing such damage. Hence, the identification of collapse in this study based on instability and localized large damage (i.e., collapse onset) aligns with criteria used in real-world structural assessment.

### Comparison and discussion

The differences in the behaviors of the models under different loading scenarios (pushover vs. dynamic), as well as the effect of the considered structural variations on the OOP responses, have been evaluated in this section, by comparing the OOP strength and damage patterns of the different numerical model variations (V1-7) against the reference post-diction models (V0).

Regarding the comparison between the pushover and dynamic OOP responses in the V0 models, the Fixed-Frame pushover led to a normalized OOP strength that closely matched the collapse PBA of the Stiff case (1.74 W in Fig. 15.a versus 1.95 g in Fig. 15.c). In contrast, the Free-Frame pushover strength (0.36 W in Fig. 15.a) was less than half of the dynamic OOP strength observed in the Flexible case (0.85 g in Fig. 15.c). Furthermore, the collapse mechanism in the Fixed-Frame pushover exhibited a one-way bending failure mode with cracking patterns that more closely align with those observed in the dynamic simulations (Fig. 10). Conversely, the Free-Frame pushover scenario primarily resulted in wall overturning, with cracks forming only during collapse (Fig. 11). These discrepancies underscored, first, the effectiveness of the Stiff dynamic loading setup in approximating the influence of a rigid diaphragm on wall response, and second, the excessive conservatism of completely disregarding the potential contribution of flexible diaphragms to the OOP dynamic stability and strength of gables.

Regarding the effects of different structural variations on the pushover and dynamic strength of the walls, a considerable increase in the Fixed- and Free-Frame OOP strengths of the models was observed as a result of implementing stronger purlin-wall connections (V1-4) up to 260% of the reference V0 models (in Fig. 15.a). However, the “dynamic” OOP resistance of the gables remained mostly unaffected by any of these configurations (in Fig. 15.c). In fact, in the no-gap V1 models, dynamic collapse occurred at similar loading intensities as the reference V0 models but with notable changes in collapse mechanisms and more brittle behaviors (Figs. 12, 13 and 14). In the static pushover loading of these cases, additional horizontal confinement provided by the vertical contacts enhanced OOP strength and stability (Figs. 10 and 11). However, during dynamic loading, while the crack patterns observed in the V0 models of Stiff (Fig. 12) and Semi-Flexible (Fig. 13) cases were delayed by the horizontal confinement, disconnection between the walls and their support and a one-way bending overturning failure occurred before this added capacity was fully mobilized. Moreover, the crack at the 2/3-height elevation of the Semi-Flexible wall (Fig. 13) was only observed during collapse. The behavior of the Flexible wall (Fig. 14) was only slightly altered in the V1 variation, specifically showing an OOP deformation 46% of its V0 counterpart and collapsing in the same manner.

The implementation of rigid purlin-wall connections in V2 variations, while increasing overall stability and static strength through the suppression of Fixed-Frame sliding (Fig. 10) and delaying the Free-Frame overturning (Fig. 11), elevated the dynamic forces transferred to the walls by eliminating the energy dissipation otherwise provided through purlin/wall sliding. Consequently, the increase in the dynamic forces resulted in more internal wall damage, manifesting as purlin detachment in the Stiff wall (Fig. 12) and two-way bending diagonal cracks initiating from the 1/3-height purlins and extending toward mid-height in the Semi-Flexible (Fig. 13) and Flexible (Fig. 14) walls. As a result, while the resistance of the Stiff wall remained unchanged (Fig. 15.c), the Semi-Flexible and Flexible walls experienced an 18% reduction in collapse PBA (collapsing one loading run earlier). Moreover, the Stiff case showed a brittle collapse at an OOP displacement equal to 10% of that of its V0 reference (Fig. 9.c), while the Semi-Flexible (Fig. 9.d) and Flexible (Fig. 9.d) showed increased deformations compared to their references due to the earlier crack development.

The aforementioned effects were further amplified in the V3 variations, where dynamic loads were transferred across a larger contact surface into the walls due to the combination of no-vertical-gap purlin-wall connections and rigid contacts. In the V3 models, while a two-way bending Fixed-Frame (Fig. 10) and multi-block one-way bending Free-Frame (Fig. 11) static failure mechanisms were observed with significantly higher strengths compared to the V0 models (with the Free-Frame model showing 67% larger initial stiffness), increased and expedited internal cracking was observed during dynamic loading (Fig. 15.b), counteracting the horizontal confinement provided by the expanded purlins and leading to reduced dynamic collapse PBAs (Fig. 15.c). Among the three walls, the highest sensitivity was demonstrated in the Flexible wall, collapsing at 57% of its reference V0 collapse PBA during the induced-seismicity portion of the loading sequence (run 6). In the Stiff and Semi-Flexible walls, reduced collapse PBAs were also observed, with maximum reductions reaching 28% relative to their V0 counterparts (Fig. 15.c).

The inclusion of anchors in the V4 models, while effectively restricting the OOP deformation of the walls under Fixed-Frame static loading (Fig. 9.a), proved ineffective in enhancing the Free-Frame static response as well as the dynamic responses. In the Free-Frame static pushover, a meaningful contribution to the OOP strength was not achieved because of the movement of the frame with the wall preventing the activation of the anchors (Fig. 11). In the dynamic simulations, the use of the anchors increased vulnerability and reduced the dynamic strength of the walls (Fig. 15.c), causing all three to collapse one loading run earlier than their V0 models (corresponding to 18% lower collapse PBAs). By restricting outward sliding of the walls away from the loading frame under dynamic loading, the use of anchors forced the Stiff (Fig. 12) and Semi-Flexible (Fig. 13) walls to collapse inward toward the loading frame. Similarly, in the Flexible wall (Fig. 14), the use of anchors introduced additional cracking beneath the top purlin, which expedited the one-way bending of the top portion of the wall. It should be noted that the variations considered in this study only slightly affected the modal response of the walls, increasing the 1st -mode frequency only by 15% (Fig. 15.a).

The reduction of the purlin-wall contact surface in V5 models only slightly reduced Free-Frame pushover strength and did not alter the Fixed-Frame static strength (Fig. 15.a), static and dynamic damage patterns (Figs. 10, 11, 12, 13 and 14), or dynamic cracking (Fig. 15.b) and collapse (Fig. 15.c) PBAs of the Semi-Flexible and Flexible walls compared to their V0 counterparts. However, higher sensitivity was exhibited in the Stiff wall (Fig. 15.c), collapsing one loading run earlier than its V0 model (corresponding to a 14% reduction in collapse PBA), with the 2/3-height crack forming at loading run 13 instead of 14 (Fig. 15.b). Since the collapse mechanism of the Stiff wall had been more sensitive to purlin-wall sliding than the other two walls, it was believed that the use of a smaller contact area had facilitated earlier purlin-wall sliding and premature collapse (Fig. 12). Moreover, in the Flexible wall (Fig. 14), while not experiencing changes in OOP resistance, an additional crack at its 1/3-height elevation was shown due to the lower confinement imposed by the bottom purlins and their detachment before collapse.

Nearly identical static and dynamic responses were observed in the pre-damaged V6 and V7 models across all three walls (Figs. 10, 11, 12, 13, 14 and 15), indicating that the use of the indirect approach of simulating pre-damage by a priori modifying the mechanical properties of the base joint (in the V7 models) was equally effective in replicating the effects of direct pre-deformation (as done in V6 models). The same static and dynamic responses as the corresponding V0 models (in terms of failure mechanism and load-displacement curves) were observed in the pre-damaged variations of both Semi-Flexible (Fig. 13) and Flexible (Fig. 14) walls. However, a minor 14% strength reduction was observed in the Free-Frame static (Fig. 15.a) and the Stiff dynamic (Fig. 15.c) walls, with collapse occurring one loading run earlier (14% lower PBA) in the latter. In Semi-Flexible and Flexible walls, the cracking of the base joint already occurred at early loading runs, and the development of the complete collapse mechanism required the formation of additional cracks that only developed at higher loading intensities. In the Free-Frame static (Fig. 10) and Stiff dynamic (Fig. 12) walls, however, earlier overturning in the former and complete sliding at the base of the latter occurred, due to the complete loss of cohesion at the base expediting failure. It should also be noted that a significantly lower initial stiffness (Fig. 9.b) was observed in the pre-damaged Free-Frame static wall compared to its V0 counterpart (26%), further underscoring the impact of the base crack on its structural response.

An interesting observation was revealed in the results of the sensitivity study: although changes in dynamic collapse mechanisms can result from using different structural configurations, the dynamic OOP acceleration capacity of the walls may only marginally be affected. This indicates that walls exhibiting different failure modes may still possess comparable OOP resistance. Another observation was made regarding the potential differences in the seismic OOP response of URM walls under static and dynamic loading, with the latter being more complex to capture through simple static loadings due to its unpredictable nature and various interactions occurring between the walls and their boundary elements under dynamic motions. However, it should also be noted that the variations considered in the current sensitivity study represented only a limited subset in the broad range of real-world structural configurations. The elimination of vertical gaps in V1 and V3 models may not have accurately reflected real construction practices and structural conditions, as small discontinuities between purlins and walls are typically present due to seasonal volumetric changes in masonry and timber. Similarly, the use of simplified anchors in V4 models may not have adequately replicated the geometry and stiffness of those applied in practice. The reduction of purlin-wall contact surfaces in the V5 models represented an upper-bound condition, which may have overestimated the extent of remaining purlin-wall contact surfaces following real seismic events. Additionally, the pre-damage level imposed in the V6 and V7 models, corresponding to a 1.2 mm crack opening, represented light pre-damage conditions (as established in (Korswagen 2024; Prosperi 2025; Giardina 2013)), whereas the introduction of more severe pre-damage levels may have potentially resulted in more pronounced degradation of wall performance. Finally, the outcomes of the sensitivity study may have been affected by the specific material properties and loading conditions of the three walls of the ERIES SUPREME campaign (Sharma et al. 2025), which had been used to calibrate the reference models. For these reasons, the findings of this section should be regarded as preliminary indicators that emphasize the need for further investigation of such configurations for a wider selection of walls and dynamic motions. The results also demonstrated the capability of the numerical modeling approach adopted in this study for such comprehensive assessments.

The use of performance-based damage states for identifying collapse has been intentionally avoided for several practical reasons. First, the progression of damage in the dynamic analyses of the sensitivity study is demonstrated in Figs. 11, 12, 13, 14 and 15 (newly added). These run-by-run identifications of new cracks serve as direct indicators of performance degradation. Since the analyses of this study represent preliminary investigations, the extension to a complete performance-based assessment has not been pursued. Second, collapse in dynamic analyses has been identified as the physical instability of the gables, and in some cases, abrupt purlin-wall detachments. In the former, the collapse of the walls was already evident by large displacements and wall-purlin detachments. In the latter cases, the damage was severe, yet highly localized at the connection zones. When evaluated using performance-based criteria such as those in EMS-98 (Grünthal 1998), the extent and critical impact of such localized damages on the global response could not be properly captured. In fact, one key takeaway of this study is the observation that although the use of strong roof-wall connections increased the nominal strength of gables, it also led, in this case, to localized damage concentrations that inhibit the effective mobilization of that strength. In other words, collapse occurred before the performance of the wall was sufficiently degraded.

The definition of performance-based damage states was also avoided due to the limitations of the current knowledge, as explained in the following. OOP collapse of URM walls under dynamic loading and experimental testing can be brittle. This behavior has been observed in this work as well as in previous experimental studies, particularly for stiffer and stronger walls, such as 2WB walls (Graziotti et al. 2019; Sharma et al. 2020), and in shake-table experiments on gables, notably the Stiff configuration of the latter (Sharma et al. 2025). Such observations contrast with previous studies based on cyclic loading (Griffith et al. 2007; Ravenshorst and Messali 2016), which, while better suited for defining damage states, indicate more ductile OOP behavior, particularly for weaker configurations such as 1WB and cantilevers (Graziotti et al. 2016). However, such tests are less representative of real earthquake loading. Consequently, a direct transition from DS0 to DS3 or even DS4/DS5 may occur, meaning a wall with minimal or no visible damage can still collapse suddenly. However, the availability of experimental and numerical evidence on the dynamic OOP response is limited. Only about ten specimens have been tested incrementally under dynamic loading using shake-table experiments, and many sources of uncertainty remain insufficiently accounted for, including unit-mortar combinations and record-to-record variability (Ghezelbash et al. 2024). Although the development of modelling strategies such as the one proposed in this paper represents an important step towards enabling virtual experiments, given the current state of the research, proposing damage states in a domain where such states are not yet well established may be premature, particularly for adoption in performance-based earthquake engineering or risk assessment frameworks.

## Conclusions

The numerical study of the out-of-plane (OOP) dynamic response of unreinforced masonry (URM) gable walls subjected to dynamic loading with different input motions representing the effect of various roof diaphragms was presented in this paper. The authors built on their contribution to the ERIES SUPREME blind prediction competition (Sharma et al. 2025), wherein the high-fidelity 3D block-based numerical model of (Ghezelbash et al. 2025; D’Altri et al. 2019) had been employed to predict the dynamic responses of gable walls tested through a multi-step sequence of shake table loading (Ghezelbash et al. 2026). In this paper, the complete experimental results, published after the competition, along with the findings of the sensitivity study conducted during the prediction phase, were used in a post-diction phase where the numerical models were updated to better reproduce the response of the two walls. The updated models were then extended to the third wall of the experimental campaign not included in the competition and further utilized to complement the ERIES SUPREME campaign by investigating different structural configurations not studied experimentally but still of scientific and engineering relevance. Specifically, the role of purlin-wall connections and pre-existing damage on the OOP behavior of gable walls was examined as these factors were considered critical to their structural performance. The following points represent the main findings:

- After minor updates during the post-diction, specifically by slightly changing the friction between purlins and walls and the tensile strength of zero-thickness joints, the numerical models captured the response of all three walls of the experimental campaign with very good quantitative and qualitative agreement. The improvements included the replication of run-by-run damage evolutions, deformation demands, interactions with the loading set-up (purlin sliding), hysteretic response, energy dissipation, and collapse mechanisms.
- In the sensitivity study, while the collapse mechanisms of walls were substantially influenced by the considered structural variations, their dynamic OOP resistance was only minimally affected. In other words, similar levels of OOP strength may correspond to different collapse mechanisms.
- Expanding the purlins to fill the vertical gaps between them and the walls, while providing horizontal confinement that prevented the cracking of the walls and increased the pushover OOP resistance of the walls, did not increase the dynamic OOP strength as the walls detached from the loading set-up before their full capacity could be mobilized.
- The introduction of strong purlin-wall connections, such as rigid ones or those with anchors, while increasing static pushover OOP strength of the specimens, failed to increase their dynamic OOP capacities. On the contrary, it intensified wall damage by transferring greater dynamic loads to them, resulting in earlier collapse at up to 18% lower dynamic loading intensities. These effects were even more pronounced when rigid connections were combined with expanded purlins, reducing the capacity by 57% in the Flexible wall and by up to 28% in the other two walls.
- Reducing the effective purlin-wall contact surfaces and introducing pre-damage had a negligible influence on the collapse mechanism of the three walls and the static and dynamic OOP resistance of the Semi-Flexible and Flexible gables. Only a 14% reduction was observed in the dynamic OOP resistance of the Stiff wall in both scenarios due to accelerated sliding at the purlin-wall connections in the first scenario and the base in the second scenario, respectively.
- The use of both approaches for simulating pre-damage, i.e., direct deformation of the loading frame and manual elimination of the cohesive strengths at the base joint, led to similar outcomes, confirming the applicability of both methods for replicating light pre-damage effects.

The improved accuracy of the numerical models, achieved through minor updating informed by the sensitivity study during the prediction phase, further demonstrated the high sensitivity of OOP dynamics to multiple parameters. Moreover, the straightforward methodology adopted for refining the numerical models based on the prediction sensitivity study showcased the robustness of the modeling approach in capturing these sensitivities. The simulation of gable walls with structural configurations beyond those assumed in the experiments demonstrated the versatility of the numerical model in complementing experimental investigations and exploring structural scenarios not covered by physical testing. The outcomes of the post-diction sensitivity study emphasized the complex influence of such structural variations on the dynamic OOP response of URM gables, particularly regarding collapse mechanisms. While providing meaningful contributions to current knowledge on URM gable dynamics, the study highlighted the need for further investigation through more extensive studies considering additional roof-wall connection strengths and higher levels of pre-damage (such as moderate and severe (Giardina 2013)).

## Data Availability

The datasets generated during the current study are available from the corresponding author on reasonable request.
